# Supplementary material for: Compassionate care intervention for hospital nursing teams caring for older people: a pilot cluster randomised controlled trial
Source: BMJ Open. 2018 Feb 22;8(2):e018563. doi: 10.1136/bmjopen-2017-018563 (PMC5879452; doi:10.1136/bmjopen-2017-018563)
Supplement: Author's manuscript [file bmjopen-2017-018563.draft_revisions.pdf]

BMJ Open is committed to open peer review. As part of this commitment we make the peer review history of every article we publish publicly available.

When an article is published we post the peer reviewers' comments and the authors' responses online. We also post the versions of the paper that were used during peer review. These are the versions that the peer review comments apply to.

The versions of the paper that follow are the versions that were submitted during the peer review process. They are not the versions of record or the final published versions. They should not be cited or distributed as the published version of this manuscript.

BMJ Open is an open access journal and the full, final, typeset and author-corrected version of record of the manuscript is available on our site with no access controls, subscription charges or pay-per-view fees (http://bmjopen.bmj.com).

If you have any questions on BMJ Open's open peer review process please email editorial.bmjopen@bmj.com

BMJ Open

Compassionate care intervention for hospital nursing teams: a pilot cluster randomised controlled trial

Journal:	*BMJ Open*
Manuscript ID	bmjopen-2017-018563
Article Type:	Research
Date Submitted by the Author:	13-Jul-2017
Complete List of Authors:	Gould, Lisa; University of Southampton Faculty of Health Sciences, University of Southampton<br>Griffiths, Peter; University of Southampton, Faculty of Health Sciences; NIHR Collaboration for Leadership in Applied Health Research and Care for Wessex<br>Barker, Hannah; University of Southampton Faculty of Health Sciences<br>Libberton, Paula; University of Southampton Faculty of Health Sciences, University of Southampton<br>Mesa-Eguiagaray, Ines; University of Southampton Faculty of Health Sciences, University of Southampton<br>Pickering, Ruth; University of Southampton Faculty of Health Sciences, University of Southampton<br>Shipway, Lisa; University of Southampton Faculty of Health Sciences, University of Southampton<br>Bridges, Jackie; University of Southampton; NIHR Collaboration for Leadership in Applied Health Research and Care for Wessex
**Primary Subject Heading**:	Health services research
Secondary Subject Heading:	Nursing
Keywords:	Compassion, Cluster randomised trial, Hospital, Nursing, Pilot, Older people

SCHOLARONE™
Manuscripts

Compassionate care intervention for hospital nursing teams: a pilot cluster randomised controlled trial

Title: Compassionate care intervention for hospital nursing teams: a pilot cluster randomised controlled trial.

Authors:

Dr Lisa Gould[1] , Professor Peter Griffiths[1,2] Miss Hannah Barker,[1] Mrs Paula Libberton[1,] Miss Ines Mesa-Eguiagaray[1,] Dr Ruth Pickering[1], Mrs Lisa Shipway[1], Professor Jackie Bridges[1,2]

Institutional affiliations of authors:

[1]Faculty of Health Sciences, University of Southampton, Southampton, UK

[2]NIHR CLAHRC Wessex, Southampton, UK

[*]**Corresponding author's contact details**: Dr Lisa Gould, University of Southampton, Faculty of Health Sciences, Nightingale Building, University Road, Highfield, Southampton, SO17 1BJ, United Kingdom l.j.gould@soton.ac.uk

Keywords: Older People, Nursing, Compassion, Cluster Randomised Trial, Pilot, Hospital,

Word count: 3812

For peer review only

Compassionate care intervention for hospital nursing teams: a pilot cluster randomised controlled trial

Abstract

OBJECTIVE: Compassionate care continues to be a focus for national and international attention, but the existing evidence base lacks the experimental methodology necessary to guide the selection of effective interventions for practice. This study aimed to evaluate the Creating Learning Environments for Compassionate Care (CLECC) intervention in improving compassionate care.

SETTING: Ward nursing teams (clusters) in two English NHS hospitals randomised to intervention (n=4) or control (n=2). Intervention wards comprised two medicines for older people (MOP) wards and two medical/surgical wards. Control wards were both MOP's.

PARTICIPANTS: Data collected from 627 patients and 178 staff. Exclusion criteria: Reverse barrier nursed, critically ill, palliative or non-English speaking. All other patients and all nursing staff and HCAs were invited to participant, agency and bank staff were excluded.

INTERVENTION: CLECC, a workplace intervention focused on developing sustainable leadership and work-team practices to support the delivery of compassionate care. Control: no educational activity.

PRIMARY AND SECONDARY OUTCOME MEASURES: Primary- Quality of Interaction Schedule (QuIS) for observed staff-patient interactions. Secondary- patient-reported evaluations of emotional care in hospital (PEECH); nurse-reported empathy (Jefferson Scale of Empathy).

RESULTS: QuIS observations achieved 93% recruitment rate with 25% of patient sample cognitively impaired. At follow-up total positive (78% versus 74%) and lower total negative (8% versus 11%) QuIS ratings favoured the direction of improvement in the intervention wards versus control wards, however these were not significant with tests of logistic regression (adjusted OR 0.03 [95% CI 0.07, 1.32]). Sixty-three percent of intervention ward patients scored lowest (i.e. more negative) scores on PEECH connection subscale, versus 79% of control, this was not statistically significant, $p \geqslant 0.05$.

CONCLUSIONS: Use of experimental methods is feasible. The use of structured observation of staff-patient interaction quality is a promising outcome measure inclusive of hard to reach groups.

Trial registration: ISRCTN16789770

Article Summary: Strengths and Limitations of this study

For peer review only - http://bmjopen.bmj.com/site/about/guidelines.xhtml

Compassionate care intervention for hospital nursing teams: a pilot cluster randomised controlled trial

- Findings from this pilot trial make an important contribution to the evidence base on the evaluation of compassionate care interventions, particularly their impact on patient-based outcomes.
- This study demonstrates that use of experimental method in this field is feasible.
- The study demonstrates where blinding was efficient, and where it was more difficult in a pragmatic hospital based intervention to control.
- Only six wards were included in this study, meaning the results may not be generalizable due to the small sample size.
- The study is of insufficient scale to draw meaningful conclusions about CLECC's effectiveness. The findings indicate, however, that more definitive evaluation is merited.

Introduction

Healthcare systems internationally are challenged by the provision of optimal care to an aging population [1]. Research into outcomes for older people admitted to hospital is far from encouraging with hospitalised older people at significant risk of functional decline [2] and frail older patients at increased risk of mortality and re-admission [3 4]. A recent systematic review on outcomes for older people in acute care suggest there is an "urgent need for the development and evaluation of effective interventions…. that optimise the care outcomes of older patients" [5]. This review found personalised treatment plans, and clear communication strategies can reduce re-admission and mortality[5]. Since person centred care and communication are features of relational care, this study aims to pilot an intervention aimed at improving relational aspects of care, i.e. capacity to experience empathy and engage in a caring relationship.

Research indicates that the quality of relationships with staff is key to shaping older people's hospital experiences, with older people valuing being seen as people, listened to and involved in treatment [6]. All of these values fall under the scope of compassionate care, requiring "relational capacity" in practitioners[7]. Thus research into what older people value in care, and conclusions from English NHS and international reports [1 8-10] suggest that interventions to improve the delivery of compassionate care are needed to meet the care needs of older people in hospitals.

For peer review only - http://bmjopen.bmj.com/site/about/guidelines.xhtml

Compassionate care intervention for hospital nursing teams: a pilot cluster randomised controlled trial

Although it is apparent that interventions to improve the delivery of compassionate care are needed, there is a lack of evidence for their efficacy, with utility limited by a seeming reluctance to use rigorous experimental methods for evaluation. A recent systematic review of evidence for compassionate nursing care interventions found that most of the 24 studies identified used uncontrolled before and after designs, with just four using randomised controlled designs [11]. Studies tended to be single-site and small-scale. A wide range of outcome measures (n=67) were deployed between the studies including staff-based outcomes (e.g. empathy), patient-based outcomes (e.g. mood) and care outcomes (e.g. patient-centeredness), indicating a lack of consensus in the field as to appropriate compassionate care outcomes and how to measure them. While most studies (79%) reported a positive effect in relation to one or more outcomes, higher quality studies were less likely to report positive effects and no interventions were evaluated more than once. Thus the quality of the evidence for effectiveness in this field is predominantly low, hampered by a lack of experimental research of sufficient scale.

Responding to an absence of high quality evidence for the effectiveness of compassionate care interventions for older patients, the study reported here aimed to pilot the use experimental methodology to evaluate a compassionate care intervention targeted at work teams in acute care settings. We aimed to provide an evidence base to guide future trial design and implementation, including feasibility of ward level randomisation, selection of outcome measures including success in blinding, sample size calculation, minimising contamination between experimental and control clusters, and maximising participation of older patients.

Methods

A multi-site pilot cluster randomised controlled trial (CRT) was undertaken with randomisation of staff and patients at ward nursing team level. Medical and surgical wards with high proportion of older patients were eligible. Six wards in two NHS hospital Trusts in England were enrolled and allocated to intervention (n=4) or control (n=2). Randomisation was undertaken using the ralloc command in Stata[12] and stratified by hospital and by ward type: Medicine for Older People (MOP) or not MOP.

For peer review only - http://bmjopen.bmj.com/site/about/guidelines.xhtml

Compassionate care intervention for hospital nursing teams: a pilot cluster randomised controlled trial

The CLECC intervention is based on workplace learning theory with the ward conceptualised as a learning environment and ward team as a community of practice[13]. It is a team-based educational programme focused on developing manager and team practices that create an expansive learning environment, theorised to enhance team capacity to provide compassionate care[14]. Expansive (rather than restrictive) environments foster workplace learning and the integration of personal and organisational development[15-17]. The intervention aims to embed ward-based manager and team practices including dialogue, reflective learning and mutual support, such that the team has the understanding and skills to continue to improve compassionate care following the end of the programmed activities. Research suggests that embedding such practises leads to a longer-term period of service improvement and sustainable improvements in practice[18]. CLECC training consisted of key activities, such as: monthly ward leader action learning sets; team learning activities, including local team climate analysis and values clarification; peer observations of practice and feedback to team by volunteer team members; team study days focused on team building and understanding patient experiences; mid-shift 5 minute team cluster discussions; and twice weekly team reflective discussions. A Practise Educator, seconded from the hospital, leads these activities. Throughout the 4-month implementation period, ward leaders and their teams developed a team-learning plan that included a patient feedback plan and measures for continuing to support leader and team practices that underpin the delivery of compassionate care. Usual practice continued on control wards.

Outcome measures were assessed at baseline (2 months before intervention and prior to randomisation to groups) and follow-up (4 months after completion of CLECC implementation period). Given anticipated patient and staff turnover between assessment periods, follow-up was at cluster level rather than individual participant level, and so recruitment for baseline and follow-up assessment periods was independent. There is no single validated measure for compassionate care so its impact was assesed across three complementary core outcomes: researcher-rated observations of the quality of staff-patient interactions, patient-reported evaluations of emotional care and nurse-reported measures of empathy. Baseline and follow-up data were also gathered on individual and ward team characteristics. We aimed to maximise the participation of patients often excluded from

For peer review only - http://bmjopen.bmj.com/site/about/guidelines.xhtml

research, that is, older people with complex needs including cognitive impairment and communication difficulties. There is insufficient literature to guide the recruitment of these groups in acute care settings, so it was not possible at the outset to predict sample size, instead more flexible target recruitment rates were used.

The quality of staff-patient interactions was assessed using the Quality of Interactions Schedule (QuIS). QuIS is a time sampling tool that measures the volume and quality of interactions through observation, enabling a calculation of how many patients experience one or more negative interactions during an observation session[19]. QuIS interactions between staff and patients are coded as positive social, positive care, neutral, negative protective or negative restrictive. Earlier piloting work has established its validity and reliability in acute settings[20].

All adult patients on participating wards were assessed for eligibility to be included in observations. Patients were excluded if they were unable to communicate their choices about taking part in the research and a consultee could not be contacted. We also excluded patients who were unconscious or where there were clinical concerns (critically ill, in receipt of palliative care, or at high infection risk). The patient sample for observations was determined by randomisation, whereby a random number generator indicated the index patient for approach from a list of eligible patients. Index patients were informed about the planned observations and if they indicated verbally or non-verbally that they were happy for the observation to proceed, other eligible patients in the researcher's field of view were approached for inclusion. If the index patient declined to take part, another index patient was randomly selected, and approached and invited as before. Staff were informed about observations with the option to withdraw if preferred. All interactions between patients and staff directly observed for two hours and coded (there were 10 x 2 hour observation sessions per ward per 3 week assessment period). Observation sessions were randomly sampled over a three weeks from Monday-Friday, 8.00 a.m.-10.00 p.m., and balanced between wards and time of day.

Patient-reported evaluations of emotional care were measured using the Patient Evaluation of Emotional Care during Hospitalisation (PEECH) survey tool[21] which is validated for use in

For peer review only - http://bmjopen.bmj.com/site/about/guidelines.xhtml

English hospital settings[21]. The subscales are security, knowledge, personal value and connection. PEECH is sensitive to changes in service quality and in ward environment[22]. All eligible patients on the ward were invited to complete a questionnaire. Patients were excluded if there were clinical concerns (as detailed above) or if they lacked capacity to consent. If recruited, patients were offered help by the researcher in completing the questionnaire.

Nurses' self-reported empathy was measured using the Jefferson Scale of Empathy (JSE)(Physician/HP version), a 20 item inventory in a 7-point Likert-type format ranging from Strongly Disagree to Strongly Agree with higher scores reflecting a more empathic orientation[23]. The JSE was developed and validated for use by health care workers, the scale is sensitive to changes in individual empathy over time and context[24 25]. All nursing staff (registered nurses and health care assistants) were invited to complete a questionnaire, based on a staff list supplied by the ward manager. Questionnaires in individually named envelopes were distributed by ward managers and returned via an on ward postbox.

A number of measures were employed to enable allocation concealment and blinding. Clusters were randomly allocated to group following baseline data collection. At follow-up, researchers conducting observations were blind to conditions. Researchers gathering questionnaire data at follow-up were aware of ward allocation. It was not possible to conceal allocation from ward team nursing staff. Patients were not informed of allocation.

All analyses were carried out on an intention to treat basis. Descriptive statistics were used to show the proportion of participants that consented to participate in study**.** The proportion of QuIS interactions rated for each of the five categories was analysed and the frequencies of patients with the lowest (most negative) scores for each subscale was calculated. The differences between groups were tested using Chi-square test. A three level mixed-effects logistic regression model was fitted to investigate the effect of the CLECC intervention on the likelihood of a negative interaction. Predictive factors were included as fixed effects and presented as odds ratios (OR) with 95% CI, after adjustment for baseline and ward consecutively. Mean PEECH and JSE scores were calculated by subscale and in total, and differences between groups at follow-up were tested using Mann-Whitney U test.

For peer review only - http://bmjopen.bmj.com/site/about/guidelines.xhtml

For peer review only

Compassionate care intervention for hospital nursing teams: a pilot cluster randomised controlled trial

In order to determine the appropriate approach for analysis and the design effect when calculating the required sample in a definitive trial, estimates of intracluster correlation were generated for each outcome measure.

Results

Six out of seven nursing ward managers invited to take part agreed to randomisation to either intervention or control. Three wards were recruited in each Trust, and all wards remained in the study until it closed, the wards had a range of 28-32 beds and mean patients stays ranged from 6days-19days. Data were collected between March 2015 and March 2016. Procedures for allocation concealment and blinding proceeded as planned, with the exception of two (out of eight) researcher observers at follow-up reporting that they learned of ward allocation from ward staff. No staff audited following observations reported that their behaviour had changed because they were being observed. Researcher field notes reflect reports from hospital managers that discussions about CLECC between staff on intervention and control wards had the potential to influence practice on the control wards, but we did not detect evidence of contamination.

Participant flow

Figure 1 shows the flow of clusters and participants through the pilot trial. Randomisation took place after baseline data collection, but results are presented by allocation for baseline and follow-up data to enable comparisons between groups.

FIGURE ONE: CONSORT flow diagram

For staff-patient observations, Figure 1 illustrates the number of approaches rather than individual patients, as some patients were invited more than once to be involved. Recruitment rate for observations at baseline was 97% (152 out of 157 approaches to eligible patients), and at follow-up was 90% (157 out of 175). Recruitment rates were similar between intervention and control wards (96% versus 98% at baseline, 90% versus 88% at follow-up). Twenty-three participants declined to participation for reasons such as, “not feeling up to it” (17%), “too unwell” (4%) and “no reason” (8%). No specific reason was recorded for 70%. In 17% (63 out of 362 approaches) the patient was approached and then assessed as not having capacity to make the decision to take part in the research. In 67%

For peer review only - http://bmjopen.bmj.com/site/about/guidelines.xhtml

(42 out of 63) of these occasions, researchers were able to contact a consultee for advice and in 100% of these cases the consultee advised that the patient should participate. A final 273 patients were observed (133 at baseline and 140 at follow-up). The mean age of patients observed was 82 years (84 years in intervention group and 77 in control). Most patients were female (77%) and 25% had evidence of cognitive impairment, with no significant differences between patients in the different experimental groups. All observation data gathered was included in analysis. Please see table 1 for Patient characteristics.

TABLE ONE

Across both assessment periods, 77% (359 out of 464) of eligible patients agreed to take part in the questionnaire survey. Overall recruitment rates were similar between intervention and control wards (77% versus 78%). Most frequent reasons recorded for patients declining participation in the questionnaire survey were "tired" (40%, n=12) and "questionnaire too difficult" (10%, n=3). The most frequent reasons recorded for excluding patients were "not having capacity" (43%, n=48) and "very cognitively impaired" (29%, n=32). Ninety nine percent (354 of 359) of patients who consented returned a completed questionnaire, with researcher help with completion given in 68% of cases. Most patients who completed questionnaires were female (70%), and aged over 70 years (83%). Of all the patient questionnaires returned, 12% were completed by patients with cognitive impairment. Intervention group patients completing questionnaires at baseline included a higher proportion of younger patients (22% aged ≤60 years versus 0%) and of males (43% versus 25%). There were no other notable differences in these characteristics between intervention and control group patients returning questionnaires (Refer to Table One for patient demographics).

Of 496 questionnaires distributed to nursing staff, 36% (n=178) were completed and returned. Return rates were more similar between baseline and follow-up (37% versus 35%). Baseline return rates were lower on intervention wards (31% versus 48%), but at follow-up were more similar between experimental groups (33% versus 39%). Most staff who returned a completed questionnaire were female (87%) and median age group was 26-35

For peer review only - http://bmjopen.bmj.com/site/about/guidelines.xhtml

years. Questionnaires were returned by 74 health care assistants (42%), 74 staff nurses (42%), and 18 sisters/charge nurses (10%), for the remaining 6% there was no data. There were no notable differences in these characteristics between intervention and control group staff returning questionnaires. All returned questionnaires (91 at baseline and 87 at follow-up) were included in analyses.

Baseline and outcome measures

As planned, 120 hours of observations took place in each assessment period, resulting in data collected on 3109 interactions between staff and patients over 240 hours. On average, each patient had 6 interactions with hospital staff per hour. Most interactions were rated as positive care (59%) and least interactions as negative protective (0.04%) for each experimental group at both assessment periods (Table 2).

TABLE TWO

At follow-up, there were higher total positive (positive social and positive care) and lower total negative (negative protective and negative restrictive) scores for intervention wards at follow-up than control wards at follow-up (78% versus 74%, 8% versus 12%). Chi square testing of these differences between groups suggested a significant difference (p=0.017). Logistic regression was used to test this result using a multi-level model to take account of clustering of observations within wards and observation sessions (Table 3). The results indicate that once other variables are taken into account in the analysis, the odds of a negative interaction are not significantly reduced because of the effect of the CLECC intervention. Results are in the direction of an effect favourable to CLECC, that is, there were less negative interactions on intervention wards, but this was not a statistically significant difference (adjusted OR 0.30 [95% CI 0.07, 1.32]).

TABLE THREE

Table 4 shows the mean patient evaluations of emotional care (PEECH) values by experimental group, together with the results of Mann-Whitney U test for differences between groups at follow-up. Higher scores indicate better patient-reported experiences.

For peer review only - http://bmjopen.bmj.com/site/about/guidelines.xhtml

Scores on the Connection subscales were consistently lower than on other subscales. Differences between experimental group at follow-up are not significant for total PEECH score or individual subscales (Mann-Whitney U, $P>0.05$), although small non-significant differences in total score and three out of four subscales favour CLECC.

TABLE FOUR

Levels of staff self-reported empathy using Jefferson Scale of Empathy was varied across the individual wards at baseline and at follow-up, A Mann-Whitney U test confirmed no significant difference between groups (p=0.800).

At ward level, ICC for QuIS, PEECH (subscales and total) and JSE was low. – ICCs were generally small (<0.027) and negligible. The ICC for QUIS at ward level was relatively higher, although still small (ICC0.071) but it was substantial at the observations session level (0.411).

Discussion:

This study aimed to deliver a compassionate care intervention in acute care settings, and pilot the use of experimental methodology in assessing the performance of selected outcome measures. We aimed to provide an evidence base to guide future trial design and implementation, including acceptability of ward level randomisation, the feasibility of assessing outcome measures, and other measures of trial implementation such as recruitment and inclusivity, sample size calculation and clustering for future trial, blinding and contamination. Our findings show that the CLECC intervention and trial randomisation is acceptable to medical and surgical nursing teams in acute care hospitals. The pilot study showed the feasibility of the sampling. All six wards remained in the study until data collection was complete, and recruitment processes and methods are inclusive of all nursing staff levels and of older patients. Patient observations were highly acceptable to patients with recruitment rates of 97% and 90%. The response rate to nursing questionnaires were somewhat low, with some larger scale studies showing European response rates of nurses to be 62%, and US nurses to be around 39% [26]. Improving staff survey response rates in future evaluations would improve confidence that bias is not skewing the questionnaire results. Piloting of these outcome measures suggests that the CLECC intervention may have

For peer review only - http://bmjopen.bmj.com/site/about/guidelines.xhtml

a favourable effect in reducing negative interactions between staff and patients, and in reducing patients' experiences of lack of emotional connection with staff. However as expected, because of the scale of this pilot, there were no significant differences once other variables were accounted for.

Hospitalised older patients with a range of cognitive abilities are a traditionally hard-to-reach group and even though they appear more prone to negative experiences of hospital care[27], they are often excluded from research [6 28 29]. It is estimated that up to 25% of beds in acute hospitals are occupied by people with dementia, with the figure likely to be higher on specialist older people's care wards [30 31]. While cognitive deficits may limit some people's ability to share their experiences, our study has been successful in devising recruitment and data collection methods that maximise support and inclusion of older people. Overall 25% of patients observed in this study had evidence of cognitive impairment, suggesting that our sample was representative of the wider hospital population. Of all the patient questionnaires returned, 12% were completed by patients with cognitive impairment, indicating the questionnaire method to be less inclusive than observational methods. Our findings echo those of Goldberg et al[28] that structured non-participant observation appears to be the most promising method to describe the experiences of older people with cognitive impairment in the general hospital setting [28].

This data gathered in this study are able to inform future observational trials in acute care settings. A particular strength is that this study accounts for inter-cluster coefficient levels, enabling a future trial design to account for clustering effects, a statistic not commonly reported in studies into healthcare interventions. The importance of this finding is that studies using observations of care should consider clustering effects when determining sample size and undertaking analysis, particularly at the observations sessions level. Additionally the study finding that contamination occurs from the cluster randomised design enables adjustments to future trial design, such as the use of stepped-wedge trial designs.

A limitation to this study is that has only been piloted on a small number of wards/ hospitals so the findings may not be generalizable, and a full future trial would best inform this. A further possible limitation of observational studies such as this is the validity of the

For peer review only - http://bmjopen.bmj.com/site/about/guidelines.xhtml

observations (in this case the QuIS ratings) as accurate representation of patient experience. Because main study observation and questionnaire data were gathered from different patient groups, it was not possible to test the validity of QuIS ratings against patient-reported experience. In our earlier feasibility work we found 79% agreement (weighted kappa 0.40: $P < 0.001$; indicating fair agreement) between patients and QuIS observers over whether interactions were positive, negative or neutral[20]. A further limitation of the QuiS rating is that being observed could, in itself, change staff behaviours. Whilst we accept this is an ever present issue with behavioural observations, both control and intervention wards are exposed to observations.

In summary the piloting of this study enhances the current body of healthcare implementation literature addressing the need for improvements in relational care and contributes to a field in which the use of experimental methodology is limited. Preliminary results from this trial suggest the outcome measures to assess relational care are feasible. This paper sets out a design for empirically evaluating a compassionate care intervention which promotes staff leadership, wellbeing and high quality care, as such linking with commitments from NHS England's 2016 framework for nursing and midwifery[32 33]. There is some debate over the use, and potential over-use, of RCT's, with as much as 85% of research investment providing losses[34], with much of this waste theorised to arise from poorly designed trials that are disconnected with real-world systems[35]. As such this intervention was designed to be pragmatic; it is primarily ward based designed to work with existing team capacity, sustainable; based on well-established theoretical models of sustainable workplace change[13], and evidence based; including this piloting work demonstrating the success of empirical evaluation of this intervention. It aligns with the goals of NHS England, and global goals for compassion[36], in particular the inclusivity of the methodology of this study allows it to addresses global concerns for compassionate care in dementia and older age[37].

Contributorship statement:

For peer review only - http://bmjopen.bmj.com/site/about/guidelines.xhtml

Compassionate care intervention for hospital nursing teams: a pilot cluster randomised controlled trial

Lisa Gould: significant contribution to design, implementation, analysis, interpretation of results, and lead write up of paper.
Jackie Bridges: lead design, implementation, analysis, interpretation of results, and contributed to write up of paper.
Peter Griffiths: significant contribution to design, analysis, interpretation of results, and write up of paper.
Ruth Pickering: significant contribution to design, analysis, interpretation of results, and reviewed write up of paper.
Ines Mesi-Eguiagaray: contribution to design, significant contribution to analysis and interpretation of results, and write up of paper.
Hannah Barker: significant contribution to design, implementation, analysis, interpretation of results, and reviewed write up of paper.
Lisa Shipway: significant contribution to implementation, analysis, interpretation of results, and reviewed write up of paper.
Paula Libberton: significant contribution to implementation, analysis, interpretation of results, and reviewed write up of paper.

Competing interests: There are no competing interests

Funding: Department of health, National Institute of Health Research, Health services and delivery research programme 13/07/48

Data sharing statement: No unpublished data is available.

References

For peer review only - http://bmjopen.bmj.com/site/about/guidelines.xhtml

Compassionate care intervention for hospital nursing teams: a pilot cluster randomised controlled trial

1. Christensen K, Doblhammer G, Rau R, et al. Ageing populations: the challenges ahead. *Lancet* 2009;374(9696):1196-208. doi: 10.1016/S0140-6736(09)61460-4
2. Huang HT, Chang CM, Liu LF, et al. Trajectories and predictors of functional decline of hospitalised older patients. *J Clin Nurs* 2013;22(9-10):1322-31. doi: 10.1111/jocn.12055
3. Karunananthan S, Wolfson C, Bergman H, et al. A multidisciplinary systematic literature review on frailty: overview of the methodology used by the Canadian Initiative on Frailty and Aging. *BMC Med Res Methodol* 2009;9:68. doi: 10.1186/1471-2288-9-68
4. Dent E, Hoogendijk EO. Psychosocial factors modify the association of frailty with adverse outcomes: a prospective study of hospitalised older people. *BMC Geriatr* 2014;14:108. doi: 10.1186/1471-2318-14-108
5. Hickman LD, Phillips JL, Newton PJ, et al. Multidisciplinary team interventions to optimise health outcomes for older people in acute care settings: A systematic review. *Arch Gerontol Geriatr* 2015;61(3):322-9. doi: 10.1016/j.archger.2015.06.021
6. Bridges J, Flatley M, Meyer J. Older people's and relatives' experiences in acute care settings: systematic review and synthesis of qualitative studies. *International Journal of Nursing Studies* 2010;47(1):89-107.
7. Bridges E, McNeill M, Munro N. Research in Review: Driving Critical Care Practice Change. *Am J Crit Care* 2016;25(1):76-84. doi: 10.4037/ajcc2016564
8. Francis R. Independent Inquiry into care provided by Mid Staffordshire NHS Foundation Trust January 2005 – March 2009. https://www.gov.uk/government/uploads/system/uploads/attachment_data/file/279109/0375_i.pdf [Accessed 18.02.2017] London: The Stationary Office 2010.
9. Francis R. Report of the Mid Staffordshire NHS Foundation Trust public inquiry. http://webarchive.nationalarchives.gov.uk/20150407084003/http://www.midstaffspublicinquiry.com/report [Accessed 18.02.2017] London, 2013.
10. Care Quality Commission. Dignity and nutrition inspection programme: national overview. https://www.cqc.org.uk/sites/default/files/documents/20111007_dignity_and_nutrition_inspection_report_final_update.pdf [Accessed 18.02.2017] Newcastle upon Tyne: Care Quality Commission, 2011.
11. Blomberg K, Griffiths P, Wengstrom Y, et al. Interventions for compassionate nursing care: a systematic review. *International Journal of Nursing Studies* 2016;62:137-55.
12. StataCorp. Stata Statistical Software: Release 12. College Station, TX: StataCorp LP, 2011.
13. Fuller A, Unwin L. Expansive learning environments: integrating organizational and personal development. In: Rainbird H, Fuller A, Munro A, eds. Workplace learning in context. London: Routledge 2004:126-44.
14. Bridges J, Fuller A. Creating Learning Environments for Compassionate Care (CLECC): a programme to promote compassionate care by health and social care teams. *International Journal of Older People Nursing* 2015;10(1):48-58.
15. Fuller A. Theories of learning and communities of practice. In: Hughes J, Jewson N, Unwin L, eds. Learning in communities of practice. London: Routledge 2007.
16. Fuller A, Unwin L. Expansive learning environments: integrating organisational and personal development. In: Rainbird H, Fuller A, Munro A, eds. Workplace learning in context. London: Routledge 2004:126-44.
17. Wenger E. Communities of practice: learning, meaning and identity. Cambridge: Cambridge University Press 1998.
18. May C, Finch T, Mair F, et al. Understanding the implementation of complex interventions in health care: the normalization process model. *BMC Health Services Research* 2007;7(1):148.
19. Dean R, Proudfoot R, Lindesay J. The quality of interactions schedule(QUIS): development, reliability and use in the evaluation of two domus units. *International Journal of Geriatric Psychiatry* 1993;8:819-26.

For peer review only - http://bmjopen.bmj.com/site/about/guidelines.xhtml

Compassionate care intervention for hospital nursing teams: a pilot cluster randomised controlled trial

20. McLean C, Griffiths P, Mesa-Eguiagaray I, et al. Reliability, feasibility, and validity of the quality of interactions schedule (QuIS) in acute hospital care: an observational study. *BMC Health Serv Res* 2017;17(1):380. doi: 10.1186/s12913-017-2312-2
21. Murrells T, Robert G, Adams M, et al. Measuring relational aspects of hospital care in England with the 'Patient Evaluation of Emotional Care during Hospitalisation'(PEECH) survey questionnaire. *BMJ Open* 2013;3(1)
22. Williams A, Dawson S, Kristjanson L. Translating theory into practice: using action research to introduce a coordinated approach to emotional care. *Patient Education and Counseling* 2008;73(1):82-90.
23. Hojat M, Mangione S, Nasca TJ, et al. The Jefferson Scale of Physician Empathy: development and preliminary psychometric data. *Educational and Psychological Measurement* 2001;61(2):349-65.
24. Hojat M, Vergare MJ, Maxwell K, et al. The devil is in the third year: a longitudinal study of erosion of empathy in medical school. *Academic Medicine* 2009;84(9):1182-91.
25. Hojat M, Mangione S, Nasca TJ, et al. An empirical study of decline in empathy in medical school. *Medical Education* 2004;38(9):934-41.
26. Aiken LH, Sermeus W, Van den Heede K, et al. Patient safety, satisfaction, and quality of hospital care: cross sectional surveys of nurses and patients in 12 countries in Europe and the United States. *BMJ: British Medical Journal* 2012;344
27. Hartley P, Gibbins N, Saunders A, et al. The association between cognitive impairment and functional outcome in hospitalised older patients: a systematic review and meta-analysis. *Age Ageing* 2017 doi: 10.1093/ageing/afx007
28. Goldberg SE, Harwood RH. Experience of general hospital care in older patients with cognitive impairment: are we measuring the most vulnerable patients' experience? *BMJ Quality & Safety* 2013;22(12):977-80.
29. van Baalen A, Vingerhoets AJJM, Sixma HJ, et al. How to evaluate quality of care from the perspective of people with dementia: an overview of the literature. *Dementia* 2010;10(1):112-37.
30. Alzheimers Society. Counting the cost: Caring for people with dementia on hospital wards. 2009
31. Mukadam N, Sampson EL. A systematic review of the prevalence, associations and outcomes of dementia in older general hospital inpatients. *International Psychogeriatrics* 2011;23(3):344-55.
32. NHS England. Leading Change Adding value,. In: NHS England, ed.: NHS England,, 2016:1-133.
33. Health Education England. Workforce plan for England: proposed education and training commissions for 2016/17. https://www.hee.nhs.uk/sites/default/files/documents/Workforce%20Plan%20for%20England%202016-17.pdf [Accessed 18.02.2017], 2016.
34. Chalmers I, Glasziou P. Avoidable waste in the production and reporting of research evidence. *Obstet Gynecol* 2009;114(6):1341-5. doi: 10.1097/AOG.0b013e3181c3020d
35. Yordanov Y, Dechartres A, Porcher R, et al. Avoidable waste of research related to inadequate methods in clinical trials. *BMJ* 2015;350:h809. doi: 10.1136/bmj.h809
36. Wortmann M. Dementia: a global health priority - highlights from an ADI and World Health Organization report. *Alzheimers Res Ther* 2012;4(5):40. doi: 10.1186/alzrt143
37. Organisation Wh, International AD. Dementia: A public health priority, 2012.

For peer review only

For peer review only - http://bmjopen.bmj.com/site/about/guidelines.xhtml

Compassionate care intervention in acute healthcare settings for older people: A pilot cluster randomised controlled trial

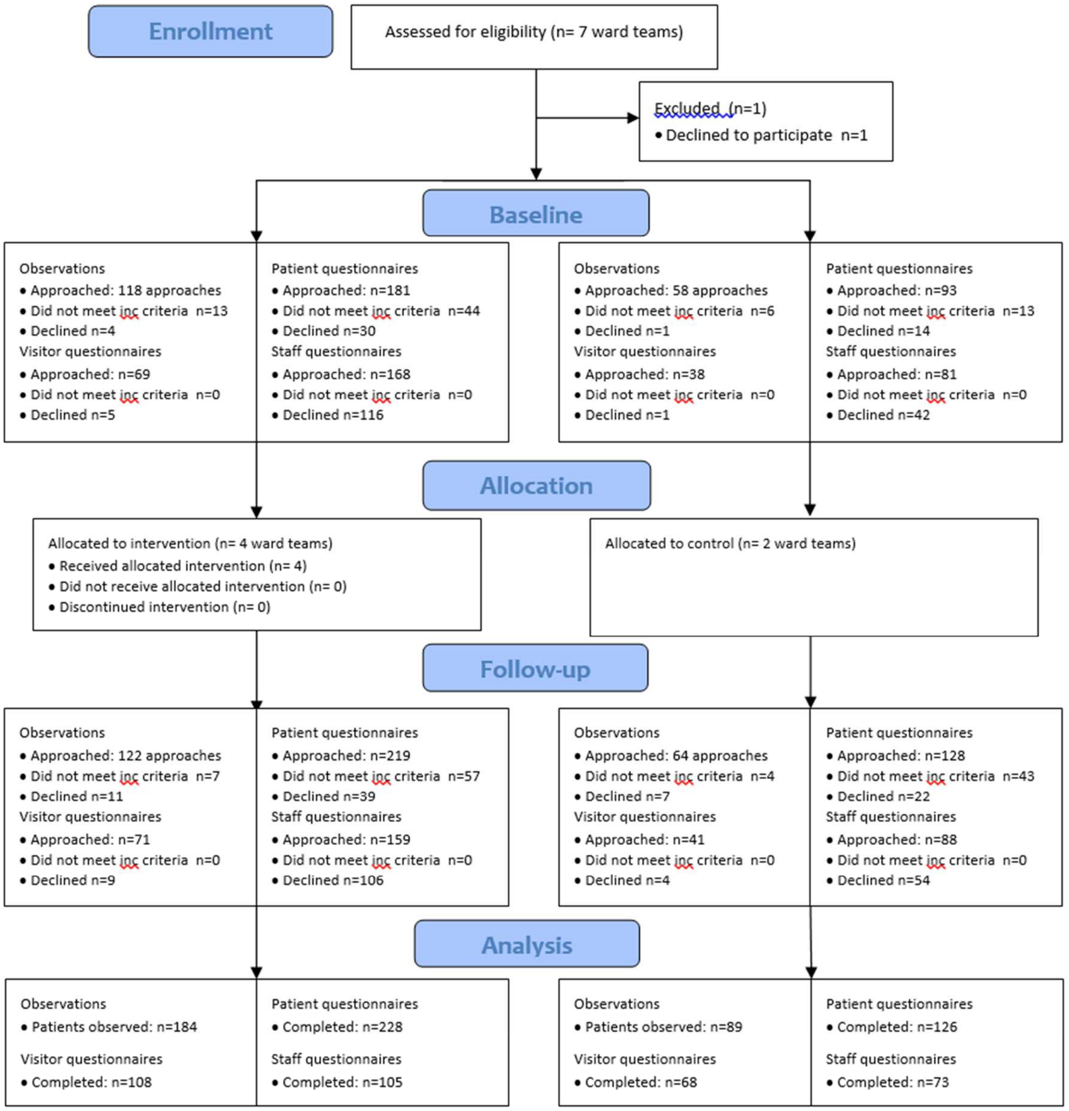

For peer review only - http://bmjopen.bmj.com/site/about/guidelines.xhtml

Compassionate care intervention in acute healthcare settings for older people: A pilot cluster randomised controlled trial

Tables For Manuscript:

Table 1: Patient Characteristics

Variable	
**Observations (n=273)**	
**Missing data=0**	
**Age**	
18-30 years	1 (0%)
31-40 years	2 (1%)
41-50 years	7 (3%)
51-60 years	14 (5%)
61-70 years	14 (5%)
More than 70 years	235 (86%)
**Gender**	
Male	63 (23%)
Female	210 (77%)
**Cognitive impairment**	
Yes	68 (25%)
No	205 (75%)
**Questionnaires (n=321)**	
**Missing data=33**	
**Age**	
18-30 years	4 (1%)
31-40 years	3 (1%)
41-50 years	9 (3%)
51-60 years	15 (5%)
61-70 years	24 (7%)
More than 70 years	266 (83%)
**Gender**	
Male	95 (30%)
Female	226 (70%)
**Cognitive impairment**	
Yes	[n=43]12%
No	[n=315] 88%
Missing	1

Table 2 Quality of staff-patient interaction QuIS by experimental group (baseline and follow-up)

	Baseline (n=1554)		Follow-up (n=1555)	
QuIS rating	CLECC (n=1143)	Control (n=411)	CLECC (n=1119)	Control (n=436)
Positive social	167 (15%)	37 (9%)	243 (22%)	64 (14%)
Positive care	672 (59%)	255 (62%)	632 (57%)	260 (60%)
Neutral	190 (17%)	77 (19%)	151 (14%)	62 (14%)

For peer review only - http://bmjopen.bmj.com/site/about/guidelines.xhtml

Compassionate care intervention in acute healthcare settings for older people: A pilot cluster randomised controlled trial

Negative protective	42 (4%)	17 (4%)	36 (3%)	21 (5%)
Negative restrictive	72 (6%)	25 (6%)	57 (5%)	29 (7%)

Table 3 QuIS multilevel logistic regression results: odds ratios (OR) of a negative interaction

**Variables**	**Model 1 unadjusted OR [95% CI] (n=3,111)**	**Model 2 adjusted OR [95% CI] (n=3,111)**	**Model 3 adjusted OR [95% CI] (n=3,111)**
CLECC effect	0.72 [0.35, 1.51]	0.47 [0.17, 1.29]	0.30 [0.07, 1.32]
Time period (Baseline vs follow-up)		0.56 [0.22, 1.43]	0.38 [0.11, 1.32]
Ward			
A			1.00
B			0.60 [0.20, 1.83]
C			0.80 [0.21, 3.05]
D			0.75 [0.24, 2.35]
E			0.61 [0.19, 1.90]
F			0.23 [0.05, 1.02]
**Variance component estimates (95% CI)**			
Observation session level (n=120)	2.13 [1.25, 3.62]	2.09 [1.23, 3.55]	1.96 [1.14, 3.37]
Patient level (n=273)	0.51 [0.23, 1.13]	0.51 [0.23, 1.13]	0.51 0.23, 1.13]

For peer review only

Table 4 PEECH mean (SD) scores by experimental group (baseline and follow-up)

PEECH	Baseline (n=168)		Follow up (n=186)		
Mean (SD)	CLECC (n=105)	Control (n=63)	CLECC (n=123)	Control (n=63)	P value
Security (0 to 3)	2.48 (0.55)	2.36 (0.51)	2.48 (0.50)	2.46 (0.48)	0.653
Knowing (0 to 3)	2.18 (0.82)	2.30 (0.72)	2.19 (0.88)	2.26 (0.66)	0.800
Personal value (0 to 3)	2.34 (0.57)	2.35 (0.58)	2.43 (0.57)	2.31 (0.57)	0.071
Connection (0 to 3)	1.68 (0.74)	1.61 (0.84)	1.81 (0.82)	1.71 (0.63)	0.350

For peer review only - http://bmjopen.bmj.com/site/about/guidelines.xhtml

Total PEECH score (0 to 66)	49.2 (11.5)	48.4 (12)	50.6 (11.3)	48.5 (9.8)	0.116

For peer review only

For peer review only - http://bmjopen.bmj.com/site/about/guidelines.xhtml

Table 2
CONSORT checklist of information to include when reporting a pilot trial

Section/topic and item No	Standard checklist item	Extension for pilot trials	Page No where item is reported
Title and abstract			
1a	Identification as a randomised trial in the title	Identification as a pilot or feasibility randomised trial in the title	Title (not allocated page number yet)
1b	Structured summary of trial design, methods, results, and conclusions (for specific guidance see CONSORT for abstracts)	Structured summary of pilot trial design, methods, results, and conclusions (for specific guidance see CONSORT abstract extension for pilot trials)	Abstract (not allocated page number yet)
Introduction			
Background and objectives:			
2a	Scientific background and explanation of rationale	Scientific background and explanation of rationale for future definitive trial, and reasons for randomised pilot trial	Pg 1-2
2b	Specific objectives or hypotheses	Specific objectives or research questions for pilot trial	Pg 2
Methods			
Trial design:			
3a	Description of trial design (such as parallel, factorial) including allocation ratio	Description of pilot trial design (such as parallel, factorial) including allocation ratio	Pg 2 and 3
3b	Important changes to methods after trial commencement (such as eligibility criteria), with	Important changes to methods after pilot trial commencement (such as eligibility criteria), with reasons	N/A, no changes made

For peer review only

Section/topic and item No	Standard checklist item	Extension for pilot trials	Page No where item is reported
	reasons		
Participants:			
4a	Eligibility criteria for participants		Pg 4 and 5
4b	Settings and locations where the data were collected		Pg 2
4c		How participants were identified and consented	Pg 3, 4 and 5
Interventions:			
5	The interventions for each group with sufficient details to allow replication, including how and when they were actually administered		Pg 3
Outcomes:			
6a	Completely defined prespecified primary and secondary outcome measures, including how and when they were assessed	Completely defined prespecified assessments or measurements to address each pilot trial objective specified in 2b, including how and when they were assessed	Pg 3, 4 and 5
6b	Any changes to trial outcomes after the trial commenced, with reasons	Any changes to pilot trial assessments or measurements after the pilot trial commenced, with reasons	N/A no changes
6c		If applicable, prespecified criteria used to judge whether, or how, to proceed with future definitive trial	

For peer review only

Section/topic and item No	Standard checklist item	Extension for pilot trials	Page No where item is reported
Sample size:			
7a	How sample size was determined	Rationale for numbers in the pilot trial	Pg 4
7b	When applicable, explanation of any interim analyses and stopping guidelines		N/A
Randomisation:			
Sequence generation:			
8a	Method used to generate the random allocation sequence		Pg 2 and 3 (ward randomisation) and 4 (observation randomisation)
8b	Type of randomisation; details of any restriction (such as blocking and block size)	Type of randomisation(s); details of any restriction (such as blocking and block size)	Pg 2 and 3, and 4
Allocation concealment mechanism:			
9	Mechanism used to implement the random allocation sequence (such as sequentially numbered containers), describing any steps taken to conceal the sequence until interventions were assigned		Pg 2 and 3,4 and 5
Implementation:			
10	Who generated the random allocation sequence, enrolled participants, and assigned participants to interventions		Pg 2, 3, 4 and 5

For peer review only

Section/topic and item No	Standard checklist item	Extension for pilot trials	Page No where item is reported
Blinding:			
11a	If done, who was blinded after assignment to interventions (eg, participants, care providers, those assessing outcomes) and how		Pg 5
11b	If relevant, description of the similarity of interventions		N/A
Analytical methods:			
12a	Statistical methods used to compare groups for primary and secondary outcomes	Methods used to address each pilot trial objective whether qualitative or quantitative	6, 7, 8 and 9
12b	Methods for additional analyses, such as subgroup analyses and adjusted analyses	Not applicable	N/A
Results			
Participant flow (a diagram is strongly recommended):			
13a	For each group, the numbers of participants who were randomly assigned, received intended treatment, and were analysed for the primary outcome	For each group, the numbers of participants who were approached and/or assessed for eligibility, randomly assigned, received intended treatment, and were assessed for each objective	Figure 1, and pg 6, 7 and 8
13b	For each group, losses and exclusions after randomisation, together with reasons		Figure 1, and pg 6, 7 and 8

For peer review only

Section/topic and item No	Standard checklist item	Extension for pilot trials	Page No where item is reported
Recruitment:			
14a	Dates defining the periods of recruitment and follow-up		Pg 6
14b	Why the trial ended or was stopped	Why the pilot trial ended or was stopped	N/A
Baseline data:			
15	A table showing baseline demographic and clinical characteristics for each group		Table 1
Numbers analysed:			
16	For each group, number of participants (denominator) included in each analysis and whether the analysis was by original assigned groups	For each objective, number of participants (denominator) included in each analysis. If relevant, these numbers should be by randomised group	Figure 1, pg 6, 7 and 8
Outcomes and estimation:			
17a	For each primary and secondary outcome, results for each group, and the estimated effect size and its precision (such as 95% confidence interval)	For each objective, results including expressions of uncertainty (such as 95% confidence interval) for any estimates. If relevant, these results should be by randomised group	Pg 8 and 9. Tables 2, 3 and 4
17b	For binary outcomes, presentation of both absolute and relative effect sizes is recommended	Not applicable	N/A
Ancillary analyses:			

For peer review only

Section/topic and item No	Standard checklist item	Extension for pilot trials	Page No where item is reported
18	Results of any other analyses performed, including subgroup analyses and adjusted analyses, distinguishing prespecified from exploratory	Results of any other analyses performed that could be used to inform the future definitive trial	N/A
Harms:			
19	All important harms or unintended effects in each group (for specific guidance see CONSORT for harms)		No harms
19a		If relevant, other important unintended consequences	N/A
Discussion			
Limitations:			
20	Trial limitations, addressing sources of potential bias, imprecision, and, if relevant, multiplicity of analyses	Pilot trial limitations, addressing sources of potential bias and remaining uncertainty about feasibility	Pg 11
Generalisability:			
21	Generalisability (external validity, applicability) of the trial findings	Generalisability (applicability) of pilot trial methods and findings to future definitive trial and other studies	Pg 10 and 11
Interpretation:			
22	Interpretation consistent with results, balancing benefits and	Interpretation consistent with pilot trial objectives and findings,	Pg 9, 10 and 11

For peer review only

Section/topic and item No	Standard checklist item	Extension for pilot trials	Page No where item is reported
	harms, and considering other relevant evidence	balancing potential benefits and harms, and considering other relevant evidence	
22a		Implications for progression from pilot to future definitive trial, including any proposed amendments	Pg 10 and 11
Other information			
Registration:			
23	Registration number and name of trial registry	Registration number for pilot trial and name of trial registry	ISRCTN16789770
Protocol:			
24	Where the full trial protocol can be accessed, if available	Where the pilot trial protocol can be accessed, if available	Submitted as supplementary file at BMJ open
Funding:			
25	Sources of funding and other support (such as supply of drugs), role of funders		NIHR HS&DR Programme
26		Ethical approval or approval by research review committee, confirmed with reference number	

For peer review only

BMJ Open

Compassionate care intervention for hospital nursing teams caring for older people: a pilot cluster randomised controlled trial

Journal:	*BMJ Open*
Manuscript ID	bmjopen-2017-018563.R1
Article Type:	Research
Date Submitted by the Author:	10-Oct-2017
Complete List of Authors:	Gould, Lisa; University of Southampton Faculty of Health Sciences, University of Southampton<br>Griffiths, Peter; University of Southampton, Faculty of Health Sciences; NIHR Collaboration for Leadership in Applied Health Research and Care for Wessex<br>Barker, Hannah; University of Southampton Faculty of Health Sciences<br>Libberton, Paula; University of Southampton Faculty of Health Sciences, University of Southampton<br>Mesa-Eguiagaray, Ines; University of Southampton Faculty of Health Sciences, University of Southampton<br>Pickering, Ruth; University of Southampton Faculty of Health Sciences, University of Southampton<br>Shipway, Lisa; University of Southampton Faculty of Health Sciences, University of Southampton<br>Bridges, Jackie; University of Southampton; NIHR Collaboration for Leadership in Applied Health Research and Care for Wessex
**Primary Subject Heading**:	Health services research
Secondary Subject Heading:	Nursing
Keywords:	Compassion, Cluster randomised trial, Hospital, Nursing, Pilot, Older people

SCHOLARONE™
Manuscripts

Compassionate care intervention for hospital nursing teams caring for older people: a pilot cluster randomised controlled trial

Compassionate care intervention for hospital nursing teams caring for older people: a pilot cluster randomised controlled trial

Authors:

Dr Lisa Gould,[1*] Professor Peter Griffiths[1,2] Ms Hannah Barker,[1] Mrs Paula Libberton,[1] Miss Ines Mesa-Eguiagaray,[1] Dr Ruth M.Pickering,[1] Shipway, Lisa, Jane[1], Professor Jackie Bridges,[1,2]

Institutional affiliations of authors:

[1]Faculty of Health Sciences, University of Southampton

[2]NIHR CLAHRC Wessex

Competing interests: none declared

Data can be requested from the corresponding author

[*]**Corresponding author's contact details**: Dr Lisa Gould, University of Southampton, Faculty of Health Sciences, Nightingale Building, University Road, Highfield, Southampton, SO17 1BJ, United Kingdom. L.j.gould@soton.ac.uk

Keywords: Compassion, Nursing, Older people, Cluster randomised trial, Hospital, Pilot

Word count: 5028 (total); 3670 (main body)

Article Summary: Strengths and Limitations of this study

- Findings from this pilot trial make an important contribution to the evidence base on the evaluation of compassionate care interventions, particularly their impact on patient-based outcomes.
- This study demonstrates that use of experimental method in this field is feasible.
- The study demonstrates where blinding was efficient, and where it was more difficult in a pragmatic hospital based intervention to control.
- Only six wards were included in this study, meaning the results may not be generalizable due to the small sample size.
- The study is of insufficient scale to draw meaningful conclusions about CLECC's effectiveness. The findings indicate, however, that more definitive evaluation is merited.

Introduction

Healthcare systems internationally are challenged by the provision of optimal care to an aging population.[1] Research into outcomes for older people admitted to hospital is far from

For peer review only - http://bmjopen.bmj.com/site/about/guidelines.xhtml

For peer review only

Compassionate care intervention for hospital nursing teams caring for older people: a pilot cluster randomised controlled trial

encouraging with hospitalised older people at significant risk of functional decline [2] and frail older patients at increased risk of mortality and re-admission.[3] A recent systematic review on outcomes for older people in acute care suggest there is an "urgent need for the development and evaluation of effective interventions…. that optimise the care outcomes of older patients".[4] This review found personalised treatment plans, and clear communication strategies can reduce re-admission and mortality.[4] This study aims to pilot an intervention aimed at improving compassionate hospital care for older people.

Research indicates that the quality of relationships with staff is key to shaping older people's hospital experiences, with older people valuing being seen as people, listened to and involved in treatment.[5] However, evidence from English NHS and international reports [1 6-8] indicates that older people frequently fail to experience positive and caring staff attitudes and behaviours, resulting in a perceived lack of compassion. Compassion is "a deep awareness of the suffering of another coupled with the wish to relieve it".[9] Being compassionate requires "relational capacity" in practitioners, i.e. capacity to experience empathy and to engage in a caring relationship.[10]

The apparent need to improve compassionate hospital care for older people has led to the development of a number of interventions, but there is a lack of evidence for their efficacy, with utility limited by a seeming reluctance to use rigorous experimental methods for evaluation. A recent systematic review of evidence for compassionate nursing care interventions found that most of the 24 studies identified used uncontrolled before and after designs, with just four using randomised controlled designs.[11] Studies tended to be single-site and small-scale. A wide range of outcome measures (n=67) were deployed between the studies including staff-based outcomes (e.g. empathy), patient-based outcomes (e.g. mood) and care outcomes (e.g. patient-centeredness), indicating a lack of consensus in the field as to appropriate compassionate care outcomes and how to measure them. While most studies (79%) reported a positive effect in relation to one or more outcomes, higher quality studies were less likely to report positive effects and no interventions were evaluated more than once. Thus the quality of the evidence for effectiveness in this field is predominantly low, hampered by a lack of experimental research of sufficient scale.

For peer review only - http://bmjopen.bmj.com/site/about/guidelines.xhtml

Compassionate care intervention for hospital nursing teams caring for older people: a pilot cluster randomised controlled trial

Responding to an absence of high quality evidence for the effectiveness of compassionate care interventions for older patients, the study reported here aimed to pilot the use experimental methodology to evaluate a compassionate care intervention targeted at work teams in acute care settings. We aimed to provide an evidence base to guide future trial design and implementation, including feasibility of ward level randomisation, selection of outcome measures including success in blinding, sample size calculation, minimising contamination between experimental and control clusters, and maximising participation of older patients.

Methods

As part of a wider feasibility study, a multi-site pilot cluster randomised controlled trial (CRT) was undertaken with randomisation of staff and patients at ward nursing team level.[12] Medical and surgical wards with high proportion of older patients were eligible. Six wards in two NHS hospital Trusts in England were enrolled and allocated to intervention (n=4) or control (n=2). Randomisation was undertaken using the ralloc command in Stata[13] and stratified by hospital and by ward type: Medicine for Older People (MOP) or not MOP.

The CLECC intervention is based on workplace learning theory with the ward conceptualised as a learning environment and ward team as a community of practice.[14] It is an educational programme focused on developing manager and team practices that create an expansive learning environment, theorised to enhance team capacity to provide compassionate care.[15] Expansive (rather than restrictive) environments foster workplace learning and the integration of personal and organisational development.[16-18] The intervention aims to embed ward-based manager and team practices including dialogue, reflective learning and mutual support. Research suggests that embedding such practices leads to a longer-term period of service improvement and sustainable improvements in practice.[19] CLECC training consisted of key activities, such as: monthly ward leader action learning sets; team learning activities, including local team climate analysis and values clarification; peer observations of practice and feedback to team by volunteer team members; team study days focused on team building and understanding patient experiences; mid-shift 5 minute team cluster discussions; and twice weekly team reflective discussions. A Practice Educator led these

For peer review only - http://bmjopen.bmj.com/site/about/guidelines.xhtml

activities through a four-month implementation period, aiming to develop a team-learning plan that included measures for continuing to support leader and team practices that underpin the delivery of compassionate care beyond the initial programmed activities. Usual practice continued on control wards. Further detail on development of the CLECC intervention can be found in Bridges and Fuller.[20]

Outcome measures were assessed at baseline (2 months before intervention and prior to randomisation to groups) and follow-up (4 months after completion of CLECC implementation period). Given anticipated patient and staff turnover between assessment periods, follow-up was at cluster level rather than individual participant level, and so recruitment for baseline and follow-up assessment periods was independent. There is no single validated measure for compassionate care so its impact was assessed across three complementary core outcomes: researcher-rated observations of the quality of staff-patient interactions, patient-reported evaluations of emotional care and nurse-reported measures of empathy. Baseline and follow-up data were also gathered on individual and ward team characteristics including patient age, cognitive impairment, ward leadership and staff turnover. We aimed to maximise the participation of older people with cognitive impairment and communication difficulties through recruitment procedures that optimised capacity to make decisions about taking part in the study.[12] Because there is insufficient literature to guide the recruitment of these groups, it was not possible at the outset to predict sample size. Instead, more flexible target recruitment rates were used.

The quality of staff-patient interactions was assessed using the Quality of Interactions Schedule (QuIS), a time sampling tool that measures the volume and quality of interactions through observation.[21] Staff-patient interactions are rated as positive social, positive care, neutral, negative protective or negative restrictive. Earlier piloting work has established its validity and reliability in acute settings.[22]

All adult patients on participating wards were assessed for eligibility to be included in observations. Patients were excluded if they were unable to communicate their choices about taking part in the research and a consultee could not be contacted. We also excluded patients who were unconscious or where there were clinical concerns (critically ill, in receipt

For peer review only - http://bmjopen.bmj.com/site/about/guidelines.xhtml

of palliative care, high infection risk). The patient sample for observations was determined by randomization of eligible patients, whereby a random number generator indicated the index patient for approach. Index patients were informed about the planned observations and if they agreed the observation could proceed, other eligible patients in the researcher's field of view were approached for inclusion. If the index patient declined to take part, another index patient was randomly selected, and approached as before. Staff were informed about observations with the option to withdraw if preferred. All interactions between patients and staff were directly observed for two hours and coded (there were 10 x 2 hour observation sessions per ward per 3 week assessment period). Observation sessions were randomly sampled over three weeks from Monday-Friday, 8.00 a.m.-10.00 p.m., and balanced between wards and time of day.

Patient-reported evaluations of emotional care were measured using the Patient Evaluation of Emotional Care during Hospitalisation (PEECH) survey tool[23] which is validated for use in English hospital settings[23]. The subscales are security, knowledge, personal value and connection. PEECH is sensitive to changes in service quality and in ward environment[24]. All eligible patients on the ward were invited to complete a questionnaire. Patients were excluded if there were clinical concerns or if they lacked capacity to consent. If recruited, patients were offered help by the researcher in completing the questionnaire.

Nurses' self-reported empathy was measured using the Jefferson Scale of Empathy (JSE)(Physician/HP version), a 20 item inventory in a 7-point Likert-type format ranging from Strongly Disagree to Strongly Agree with higher scores reflecting a more empathic orientation[25]. The JSE was developed and validated for use by health care workers, the scale is sensitive to changes in individual empathy over time and context[26 27]. All nursing staff (registered nurses and health care assistants) were invited to complete a questionnaire, based on a staff list supplied by the ward manager. Questionnaires in individually named envelopes were distributed by ward managers and returned via an on-ward postbox.

A number of measures were employed to enable allocation concealment and blinding. Clusters were randomly allocated to group following baseline data collection. At follow-up, researchers conducting observations were blinded to allocation, but researchers gathering

For peer review only - http://bmjopen.bmj.com/site/about/guidelines.xhtml

questionnaire data were aware of ward allocation. It was not possible to conceal allocation from ward team nursing staff. Patients were not informed of allocation.

All analyses were carried out on an intention to treat basis. Descriptive statistics were used to show the proportion of participants that consented to participate in study**.** The proportion of QuIS interactions rated for each of the five categories was analysed and the frequencies of patients with the lowest (most negative) scores for each subscale was calculated. The differences between groups were tested using Chi-square test. A three level mixed-effects logistic regression model was fitted to investigate the effect of the CLECC intervention on the likelihood of a negative interaction. Predictive factors were included as fixed effects and presented as odds ratios (OR) with 95% CI, after adjustment for baseline and ward consecutively. Mean PEECH and JSE scores were calculated by subscale and in total, and differences between groups at follow-up were tested using Mann-Whitney U test. In order to determine the appropriate approach for analysis and the design effect when calculating the required sample in a definitive trial, estimates of intracluster correlation were generated for each outcome measure.

Results

Six out of seven nursing ward managers invited to take part agreed to randomisation to either intervention or control. Three wards were recruited in each Trust, and all wards remained in the study until it closed, the wards had a range of 28-32 beds and mean patients stays ranged from 6days-19days. Data were collected between March 2015 and March 2016. Procedures for allocation concealment and blinding proceeded as planned, with the exception of two (out of eight) researcher observers at follow-up reporting that they learned of ward allocation from ward staff. No staff audited following observations reported that their behaviour had changed because they were being observed. Researcher field notes reflect reports from hospital managers that discussions about CLECC between staff on intervention and control wards had the potential to influence practice on the control wards, but we did not detect evidence of contamination.

Participant flow

For peer review only - http://bmjopen.bmj.com/site/about/guidelines.xhtml

Compassionate care intervention for hospital nursing teams caring for older people: a pilot cluster randomised controlled trial

Figure 1 shows the flow of clusters and participants through the pilot trial. Randomisation took place after baseline data collection, but results are presented by allocation for baseline and follow-up data to enable comparisons between groups.

FIGURE ONE: CONSORT flow diagram

For staff-patient observations, Figure 1 illustrates the number of approaches rather than individual patients, as some patients were invited more than once to be involved. Recruitment rate for observations at baseline was 97% (152 out of 157 approaches to eligible patients), and at follow-up was 90% (157 out of 175). Recruitment rates were similar between intervention and control wards (96% versus 98% at baseline, 90% versus 88% at follow-up). Twenty-three participants declined to participate for reasons including “not feeling up to it” (17%), or “too unwell” (4%). No specific reason was recorded for 70%. In 17% (63 out of 362 approaches) patients were assessed as not having capacity to make the decision to take part. In 67% (42 out of 63) of these occasions, researchers were able to contact a consultee for advice and in 100% of these cases the consultee advised that the patient should participate. A final 273 patients were observed (133 at baseline and 140 at follow-up). The mean age of patients observed was 82 years (84 years in intervention group and 77 in control) (Table 1). Most patients were female (77%) and 25% had evidence of cognitive impairment, with no significant differences by experimental group. All observation data gathered were included in analysis.

Variable	
**Observations (n=273)**	
**Missing data=0**	
**Age**	
18-30 years	1 (0%)
31-40 years	2 (1%)
41-50 years	7 (3%)
51-60 years	14 (5%)
61-70 years	14 (5%)
More than 70 years	235 (86%)
**Gender**	
Male	63 (23%)
Female	210 (77%)
**Cognitive impairment**	

For peer review only - http://bmjopen.bmj.com/site/about/guidelines.xhtml

Compassionate care intervention for hospital nursing teams caring for older people: a pilot cluster randomised controlled trial

Yes	68 (25%)
No	205 (75%)
**Questionnaires (n=321)**	
**Missing data=33**	
**Age**	
18-30 years	4 (1%)
31-40 years	3 (1%)
41-50 years	9 (3%)
51-60 years	15 (5%)
61-70 years	24 (7%)
More than 70 years	266 (83%)
**Gender**	
Male	95 (30%)
Female	226 (70%)
**Cognitive impairment**	
Yes	[n=43]12%
No	[n=315] 88%
Missing	1

Table 1: Patient Characteristics

Across both assessment periods, 77% (359 out of 464) of eligible patients agreed to take part in the questionnaire survey. Overall recruitment rates were similar between intervention and control wards (77% versus 78%). Most frequent reasons recorded for patients declining participation in the questionnaire survey were "tired" (40%, n=12) and "questionnaire too difficult" (10%, n=3). The most frequent reasons recorded for excluding patients were "not having capacity" (43%, n=48) and "very cognitively impaired" (29%, n=32). Ninety nine percent (354 of 359) of patients who consented returned a completed questionnaire, with researcher help with completion given in 68% of cases. Most patients were female (70%), and aged over 70 years (83%). Twelve percent of patient questionnaires were completed by patients with cognitive impairment. Intervention group patients completing questionnaires at baseline included a higher proportion of younger patients (22% aged ≤60 years versus 0%) and of males (43% versus 25%). There were no other notable differences by experimental group (Table 1).

Of 496 questionnaires distributed to nursing staff, 36% (n=178) were completed and returned (37% at baseline, 35% at follow-up). Baseline return rates were lower on

For peer review only - http://bmjopen.bmj.com/site/about/guidelines.xhtml

intervention wards (31% versus 48%), but at follow-up were more similar between experimental groups (33% versus 39%). Most staff who returned a completed questionnaire were female (87%) and median age group was 26-35 years. Questionnaires were returned by 74 health care assistants (42%), 74 staff nurses (42%), and 18 sisters/charge nurses (10%), (missing data=6%). There were no notable differences in job role by experimental group. All returned questionnaires (91 at baseline and 87 at follow-up) were included in analyses.

Baseline and outcome measures

As planned, 120 hours of observations took place in each assessment period, resulting in data collected on 3109 interactions between staff and patients over 240 hours. On average, each patient had 6 interactions with hospital staff per hour. Most interactions were rated as positive care (59%) and least interactions as negative protective (4%) for each experimental group at both assessment periods (Table 2).

	Baseline (n=1554)		Follow-up (n=1555)	
QuIS rating	CLECC (n=1143)	Control (n=411)	CLECC (n=1119)	Control (n=436)
Positive social	167 (15%)	37 (9%)	243 (22%)	64 (14%)
Positive care	672 (59%)	255 (62%)	632 (57%)	260 (60%)
Neutral	190 (17%)	77 (19%)	151 (14%)	62 (14%)
Negative protective	42 (4%)	17 (4%)	36 (3%)	21 (5%)
Negative restrictive	72 (6%)	25 (6%)	57 (5%)	29 (7%)

Table 2 Quality of staff-patient interaction QuIS by experimental group (baseline and follow-up)

At follow-up, there were higher total positive (positive social and positive care) and lower total negative (negative protective and negative restrictive) scores for intervention wards than control (78% versus 74%, 8% versus 12%). Chi square testing suggested these differences were significant (p=0.017). However, multilevel logistic regression results

For peer review only - http://bmjopen.bmj.com/site/about/guidelines.xhtml

indicate that once other variables are taken into account, the odds of a negative interaction are not significantly reduced because of the effect of the CLECC intervention (Table 3). Results are in the direction of an effect favourable to CLECC, that is, there were less negative interactions on intervention wards, but this is not a statistically significant difference (adjusted OR 0.30 [95% confidence interval 0.07, 1.32]).

**Variables**	**Model 1 unadjusted OR [95% CI] (n=3,111)**	**Model 2 adjusted OR [95% CI] (n=3,111)**	**Model 3 adjusted OR [95% CI] (n=3,111)**
CLECC effect	0.72 [0.35, 1.51]	0.47 [0.17, 1.29]	0.30 [0.07, 1.32]
Time period (Baseline vs follow-up)		0.56 [0.22, 1.43]	0.38 [0.11, 1.32]
Ward			
A			1.00
B			0.60 [0.20, 1.83]
C			0.80 [0.21, 3.05]
D			0.75 [0.24, 2.35]
E			0.61 [0.19, 1.90]
F			0.23 [0.05, 1.02]
**Variance component estimates (95% CI)**			
Observation session level (n=120)	2.13 [1.25, 3.62]	2.09 [1.23, 3.55]	1.96 [1.14, 3.37]
Patient level (n=273)	0.51 [0.23, 1.13]	0.51 [0.23, 1.13]	0.51 0.23, 1.13]

Table 3 QuIS multilevel logistic regression results: odds ratios (OR) of a negative interaction

Table 4 shows the mean patient evaluations of emotional care (PEECH) values by experimental group. Higher scores indicate better patient-reported experiences. Connection subscale scores were consistently lower than on other subscales. Differences between groups at follow-up favor CLECC in total score and three of the four subscales, but these differences were not significant.

For peer review only - http://bmjopen.bmj.com/site/about/guidelines.xhtml

Compassionate care intervention for hospital nursing teams caring for older people: a pilot cluster randomised controlled trial

PEECH Mean (SD)	Baseline (n=168)		Follow up (n=186)		P value
	CLECC (n=105)	Control (n=63)	CLECC (n=123)	Control (n=63)	
Security (0 to 3)	2.48 (0.55)	2.36 (0.51)	2.48 (0.50)	2.46 (0.48)	0.653
Knowing (0 to 3)	2.18 (0.82)	2.30 (0.72)	2.19 (0.88)	2.26 (0.66)	0.800
Personal value (0 to 3)	2.34 (0.57)	2.35 (0.58)	2.43 (0.57)	2.31 (0.57)	0.071
Connection (0 to 3)	1.68 (0.74)	1.61 (0.84)	1.81 (0.82)	1.71 (0.63)	0.350
Total PEECH score (0 to 66)	49.2 (11.5)	48.4 (12)	50.6 (11.3)	48.5 (9.8)	0.116

Table 4 PEECH mean (SD) scores by experimental group (baseline and follow-up)

Levels of staff self-reported empathy using Jefferson Scale of Empathy varied across individual wards at baseline and at follow-up. There was no significant difference between groups (p=0.800).

At ward level, intracluster correlations (ICCs) for QuIS, PEECH and JSE were low (<0.027). The ICC for QuIS at ward level was higher, although still small (0.071), but high at observation session level (0.411).

Discussion:

This study aimed to deliver a compassionate care intervention in acute care settings, and pilot the use of experimental methodology in assessing the performance of selected outcome measures. We aimed to provide an evidence base to guide future trial design and implementation, including acceptability of ward level randomisation, the feasibility of assessing outcome measures, and other measures of trial implementation such as recruitment and inclusivity, sample size calculation and clustering for future trial, blinding and contamination. Our findings show that the CLECC intervention and trial randomisation is acceptable to medical and surgical nursing teams in acute care hospitals. The pilot study showed the feasibility of the sampling. All six wards remained in the study until data collection was complete, and recruitment processes and methods are inclusive of all nursing staff levels and of older patients. Patient observations were highly acceptable to patients

For peer review only - http://bmjopen.bmj.com/site/about/guidelines.xhtml

with recruitment rates of 97% and 90%. The response rate to nursing questionnaires were somewhat low, with some larger scale studies showing European response rates of nurses to be 62%, and US nurses to be around 39% [28]. Improving staff survey response rates through further feasibility work would improve confidence that bias is not skewing the questionnaire results. Piloting of these outcome measures suggests that the CLECC intervention may have a favourable effect in reducing negative interactions between staff and patients, and in reducing patients' experiences of lack of emotional connection with staff. However as expected, because of the scale of this pilot, there were no significant differences once other variables were accounted for.

Hospitalised older patients with cognitive impairment are a traditionally hard-to-reach group and even though they appear more prone to negative experiences of hospital care,[29] they are often excluded from research.[5 30 31] It is estimated that up to 25% of beds in acute hospitals are occupied by people with dementia, with the figure likely to be higher on specialist older people's wards.[32 33] While cognitive deficits may limit some people's ability to share their experiences, our study has been successful in devising recruitment and data collection methods that maximise their inclusion. Overall 25% of patients observed in this study had evidence of cognitive impairment, suggesting a sample representative of the wider hospital population. Twelve percent of patient questionnaires returned were completed by patients with cognitive impairment, indicating the questionnaire method was inclusive than observation methods. Participating in an observation does not require any particular state of health, abilities or performance form the patient in question, whereas participating in a questionnaire about one's care experiences requires a minimum orientation to place, language skills and attention.[31] In addition, using questionnaire methods may be psychologically threatening to patients still in receipt of care, regardless of cognitive status.[34]

The validity of observer ratings as accurate representation of patient experiences merits attention. Because main study observation and questionnaire data were gathered from different patient groups, it was not possible to test the validity of observer ratings against patient-reported experience. However, in earlier piloting work we found 79% agreement (weighted kappa 0.40: $P < 0.001$; indicating fair agreement) between patients' and

For peer review only - http://bmjopen.bmj.com/site/about/guidelines.xhtml

observers' ratings of interaction quality.[22] Our findings echo those of Goldberg et al.[30] that structured non-participant observation appears to be the most promising method to describe the experiences of older people with cognitive impairment in the general hospital setting.[30]

These findings can inform future trials in acute care settings, a particular strength being the report of intracluster correlation, a statistic not commonly reported in studies into healthcare interventions. A clear design effect was apparent with QuIS at observation session level and this can inform future trial design. Additionally the finding of possible contamination between wards can inform future trial design, and the use of designs such as stepped-wedge may be indicated here.

This study was piloted on a small number of wards in two hospitals so the findings may not be generalizable. In addition, being observed could, in itself, change staff behaviours, and a common limitation of trials of this kind when it is not possible to conceal allocation from staff, is that bias may influence staff responses to observations and questionnaires. Finally our blinding strategies for follow-up observers require further development for a future study.

Findings from our wider study, reported elsewhere, that implementation of the CLECC intervention was uneven between wards, difficult to sustain and dependent on organisational support,[35] indicate that, while experimental research in this field is necessary, it will not provide sufficient explanation of results if conducted in isolation. However, the findings reported here represent valuable groundwork to the further development of sound experimental design in a field in which good design and implementation are very much needed.

Contributorship statement:

For peer review only - http://bmjopen.bmj.com/site/about/guidelines.xhtml

Compassionate care intervention for hospital nursing teams caring for older people: a pilot cluster randomised controlled trial

Lisa Gould: significant contribution to design, implementation, analysis, interpretation of results, and lead write up of paper.
Jackie Bridges: lead design, implementation, analysis, interpretation of results, and contributed to write up of paper.
Peter Griffiths: significant contribution to design, analysis, interpretation of results, and write up of paper.
Ruth Pickering: significant contribution to design, analysis, interpretation of results, and reviewed write up of paper.
Ines Mesi-Eguiagaray: contribution to design, significant contribution to analysis and interpretation of results, and write up of paper.
Hannah Barker: significant contribution to design, implementation, analysis, interpretation of results, and reviewed write up of paper.
Lisa Shipway: significant contribution to implementation, analysis, interpretation of results, and reviewed write up of paper.
Paula Libberton: significant contribution to implementation, analysis, interpretation of results, and reviewed write up of paper.

Funding: Department of health, National Institute of Health Research, Health services and delivery research programme 13/07/48

References

For peer review only

Compassionate care intervention for hospital nursing teams caring for older people: a pilot cluster randomised controlled trial

1. Christensen K, Doblhammer G, Rau R, et al. Ageing populations: the challenges ahead. *Lancet* 2009;374(9696):1196-208. doi: 10.1016/S0140-6736(09)61460-4
2. Huang HT, Chang CM, Liu LF, et al. Trajectories and predictors of functional decline of hospitalised older patients. *J Clin Nurs* 2013;22(9-10):1322-31. doi: 10.1111/jocn.12055
3. Dent E, Chapman I, Howell S, et al. Frailty and functional decline indices predict poor outcomes in hospitalised older people. *Age Ageing* 2014;43(4):477-84. doi: 10.1093/ageing/aft181
4. Hickman LD, Phillips JL, Newton PJ, et al. Multidisciplinary team interventions to optimise health outcomes for older people in acute care settings: A systematic review. *Arch Gerontol Geriatr* 2015;61(3):322-9. doi: 10.1016/j.archger.2015.06.021
5. Bridges J, Flatley M, Meyer J. Older people's and relatives' experiences in acute care settings: systematic review and synthesis of qualitative studies. *International Journal of Nursing Studies* 2010;47(1):89-107.
6. Francis R. Independent Inquiry into care provided by Mid Staffordshire NHS Foundation Trust January 2005 – March 2009. https://www.gov.uk/government/uploads/system/uploads/attachment_data/file/279109/0375_i.pdf [Accessed 18.02.2017] London: The Stationary Office 2010.
7. Francis R. Report of the Mid Staffordshire NHS Foundation Trust public inquiry. http://webarchive.nationalarchives.gov.uk/20150407084003/http://www.midstaffspublicinquiry.com/report [Accessed 18.02.2017] London, 2013.
8. Care Quality Commission. Dignity and nutrition inspection programme: national overview. https://www.cqc.org.uk/sites/default/files/documents/20111007_dignity_and_nutrition_inspection_report_final_update.pdf [Accessed 18.02.2017] Newcastle upon Tyne: Care Quality Commission, 2011.
9. Chochinov H. Dignity and the essence of medicine: the A, B, C, and D of dignity conserving care. *British Medical Journal* 2007;335:184-87.
10. Hartrick G. Relational capacity: the foundation for interpersonal nursing practice. *Journal of Advanced Nursing* 1997;26(3):523-28.
11. Blomberg K, Griffiths P, Wengstrom Y, et al. Interventions for compassionate nursing care: a systematic review. *International Journal of Nursing Studies* 2016;62:137-55.
12. Barker H, Griffiths P, Gould L, et al. Quantity and quality of interaction between staff and older patients in UK hospital wards: a descriptive study. *International Journal of Nursing Studies* 2016;62:100-07.
13. StataCorp. Stata Statistical Software: Release 12. College Station, TX: StataCorp LP, 2011.
14. Fuller A, Unwin L. Expansive learning environments: integrating organizational and personal development. In: Rainbird H, Fuller A, Munro A, eds. Workplace learning in context. London: Routledge 2004:126-44.
15. Bridges J, Fuller A. Creating Learning Environments for Compassionate Care (CLECC): a programme to promote compassionate care by health and social care teams. *International Journal of Older People Nursing* 2015;10(1):48-58.
16. Fuller A. Theories of learning and communities of practice. In: Hughes J, Jewson N, Unwin L, eds. Learning in communities of practice. London: Routledge 2007.
17. Fuller A, Unwin L. Expansive learning environments: integrating organisational and personal development. In: Rainbird H, Fuller A, Munro A, eds. Workplace learning in context. London: Routledge 2004:126-44.
18. Wenger E. Communities of practice: learning, meaning and identity. Cambridge: Cambridge University Press 1998.
19. May C, Finch T, Mair F, et al. Understanding the implementation of complex interventions in health care: the normalization process model. *BMC Health Services Research* 2007;7(1):148.

For peer review only - http://bmjopen.bmj.com/site/about/guidelines.xhtml

Compassionate care intervention for hospital nursing teams caring for older people: a pilot cluster randomised controlled trial

20. Bridges J, Fuller A. Creating learning environments for compassionate care: a programme to promote compassionate care by health and social care teams. *Int J Older People Nurs* 2015;10(1):48-58. doi: 10.1111/opn.12055
21. Dean R, Proudfoot R, Lindesay J. The quality of interactions schedule(QUIS): development, reliability and use in the evaluation of two domus units. *International Journal of Geriatric Psychiatry* 1993;8:819-26.
22. McLean C, Griffiths P, Mesa-Eguiagaray I, et al. Reliability, feasibility, and validity of the quality of interactions schedule (QuIS) in acute hospital care: an observational study. *BMC Health Serv Res* 2017;17(1):380. doi: 10.1186/s12913-017-2312-2
23. Murrells T, Robert G, Adams M, et al. Measuring relational aspects of hospital care in England with the 'Patient Evaluation of Emotional Care during Hospitalisation'(PEECH) survey questionnaire. *BMJ Open* 2013;3(1)
24. Williams A, Dawson S, Kristjanson L. Translating theory into practice: using action research to introduce a coordinated approach to emotional care. *Patient Education and Counseling* 2008;73(1):82-90.
25. Hojat M, Mangione S, Nasca TJ, et al. The Jefferson Scale of Physician Empathy: development and preliminary psychometric data. *Educational and Psychological Measurement* 2001;61(2):349-65.
26. Hojat M, Vergare MJ, Maxwell K, et al. The devil is in the third year: a longitudinal study of erosion of empathy in medical school. *Academic Medicine* 2009;84(9):1182-91.
27. Hojat M, Mangione S, Nasca TJ, et al. An empirical study of decline in empathy in medical school. *Medical Education* 2004;38(9):934-41.
28. Aiken LH, Sermeus W, Van den Heede K, et al. Patient safety, satisfaction, and quality of hospital care: cross sectional surveys of nurses and patients in 12 countries in Europe and the United States. *BMJ: British Medical Journal* 2012;344
29. Hartley P, Gibbins N, Saunders A, et al. The association between cognitive impairment and functional outcome in hospitalised older patients: a systematic review and meta-analysis. *Age Ageing* 2017 doi: 10.1093/ageing/afx007
30. Goldberg SE, Harwood RH. Experience of general hospital care in older patients with cognitive impairment: are we measuring the most vulnerable patients' experience? *BMJ Quality & Safety* 2013;22(12):977-80.
31. van Baalen A, Vingerhoets AJJM, Sixma HJ, et al. How to evaluate quality of care from the perspective of people with dementia: an overview of the literature. *Dementia* 2010;10(1):112-37.
32. Alzheimers Society. Counting the cost: Caring for people with dementia on hospital wards. 2009
33. Mukadam N, Sampson EL. A systematic review of the prevalence, associations and outcomes of dementia in older general hospital inpatients. *International Psychogeriatrics* 2011;23(3):344-55.
34. La Monica EL, Wolf RM, Madea AR, et al. Empathy and nursing care outcomes. *Scholarly Inquiry for Nursing Practice* 1987;1(3):197-213.
35. Bridges J, May C, Fuller A, et al. Optimising impact and sustainability: a qualitative process evaluation of a complex intervention targeted at compassionate care. *BMJ Qual Saf* 2017 doi: 10.1136/bmjqs-2017-006702

For peer review only - http://bmjopen.bmj.com/site/about/guidelines.xhtml

CONSORT Participant Flow Diagram for Pilot Cluster RCT

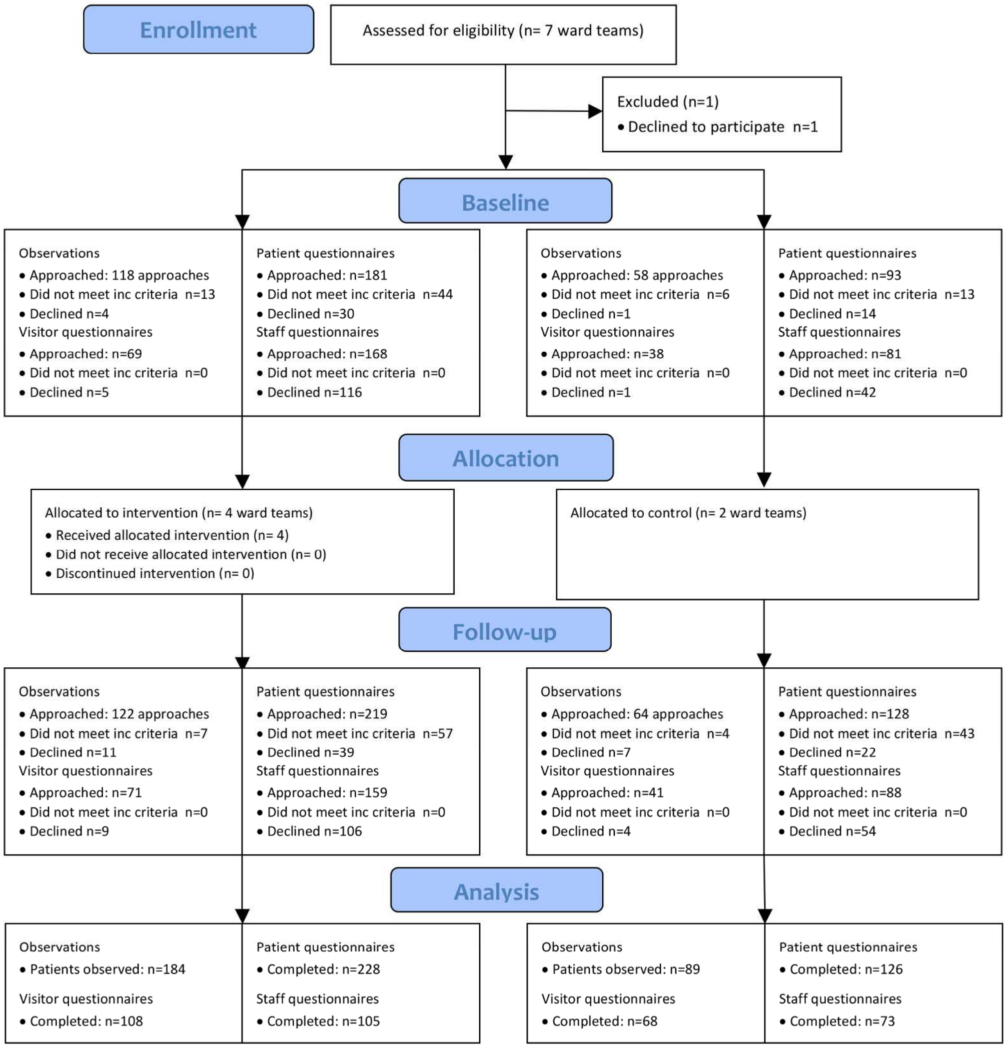

187x222mm (300 x 300 DPI)

Table 2
CONSORT checklist of information to include when reporting a pilot trial

Section/topic and item No	Standard checklist item	Extension for pilot trials	Page No where item is reported
Title and abstract			
1a	Identification as a randomised trial in the title	Identification as a pilot or feasibility randomised trial in the title	Title (not allocated page number yet)
1b	Structured summary of trial design, methods, results, and conclusions (for specific guidance see CONSORT for abstracts)	Structured summary of pilot trial design, methods, results, and conclusions (for specific guidance see CONSORT abstract extension for pilot trials)	Abstract (not allocated page number yet)
Introduction			
Background and objectives:			
2a	Scientific background and explanation of rationale	Scientific background and explanation of rationale for future definitive trial, and reasons for randomised pilot trial	Pg 1-2
2b	Specific objectives or hypotheses	Specific objectives or research questions for pilot trial	Pg 2
Methods			
Trial design:			
3a	Description of trial design (such as parallel, factorial) including allocation ratio	Description of pilot trial design (such as parallel, factorial) including allocation ratio	Pg 2 and 3
3b	Important changes to methods after trial commencement (such as eligibility criteria), with	Important changes to methods after pilot trial commencement (such as eligibility criteria), with reasons	N/A, no changes made

For peer review only

Section/topic and item No	Standard checklist item	Extension for pilot trials	Page No where item is reported
	reasons		
Participants:			
4a	Eligibility criteria for participants		Pg 4 and 5
4b	Settings and locations where the data were collected		Pg 2
4c		How participants were identified and consented	Pg 3, 4 and 5
Interventions:			
5	The interventions for each group with sufficient details to allow replication, including how and when they were actually administered		Pg 3
Outcomes:			
6a	Completely defined prespecified primary and secondary outcome measures, including how and when they were assessed	Completely defined prespecified assessments or measurements to address each pilot trial objective specified in 2b, including how and when they were assessed	Pg 3, 4 and 5
6b	Any changes to trial outcomes after the trial commenced, with reasons	Any changes to pilot trial assessments or measurements after the pilot trial commenced, with reasons	N/A no changes
6c		If applicable, prespecified criteria used to judge whether, or how, to proceed with future definitive trial	

For peer review only

Section/topic and item No	Standard checklist item	Extension for pilot trials	Page No where item is reported
Sample size:			
7a	How sample size was determined	Rationale for numbers in the pilot trial	Pg 4
7b	When applicable, explanation of any interim analyses and stopping guidelines		N/A
Randomisation:			
Sequence generation:			
8a	Method used to generate the random allocation sequence		Pg 2 and 3 (ward randomisation) and 4 (observation randomisation)
8b	Type of randomisation; details of any restriction (such as blocking and block size)	Type of randomisation(s); details of any restriction (such as blocking and block size)	Pg 2 and 3, and 4
Allocation concealment mechanism:			
9	Mechanism used to implement the random allocation sequence (such as sequentially numbered containers), describing any steps taken to conceal the sequence until interventions were assigned		Pg 2 and 3,4 and 5
Implementation:			
10	Who generated the random allocation sequence, enrolled participants, and assigned participants to interventions		Pg 2, 3, 4 and 5

For peer review only

Section/topic and item No	Standard checklist item	Extension for pilot trials	Page No where item is reported
Blinding:			
11a	If done, who was blinded after assignment to interventions (eg, participants, care providers, those assessing outcomes) and how		Pg 5
11b	If relevant, description of the similarity of interventions		N/A
Analytical methods:			
12a	Statistical methods used to compare groups for primary and secondary outcomes	Methods used to address each pilot trial objective whether qualitative or quantitative	6, 7, 8 and 9
12b	Methods for additional analyses, such as subgroup analyses and adjusted analyses	Not applicable	N/A
Results			
Participant flow (a diagram is strongly recommended):			
13a	For each group, the numbers of participants who were randomly assigned, received intended treatment, and were analysed for the primary outcome	For each group, the numbers of participants who were approached and/or assessed for eligibility, randomly assigned, received intended treatment, and were assessed for each objective	Figure 1, and pg 6, 7 and 8
13b	For each group, losses and exclusions after randomisation, together with reasons		Figure 1, and pg 6, 7 and 8

For peer review only

Section/topic and item No	Standard checklist item	Extension for pilot trials	Page No where item is reported
Recruitment:			
14a	Dates defining the periods of recruitment and follow-up		Pg 6
14b	Why the trial ended or was stopped	Why the pilot trial ended or was stopped	N/A
Baseline data:			
15	A table showing baseline demographic and clinical characteristics for each group		Table 1
Numbers analysed:			
16	For each group, number of participants (denominator) included in each analysis and whether the analysis was by original assigned groups	For each objective, number of participants (denominator) included in each analysis. If relevant, these numbers should be by randomised group	Figure 1, pg 6, 7 and 8
Outcomes and estimation:			
17a	For each primary and secondary outcome, results for each group, and the estimated effect size and its precision (such as 95% confidence interval)	For each objective, results including expressions of uncertainty (such as 95% confidence interval) for any estimates. If relevant, these results should be by randomised group	Pg 8 and 9. Tables 2, 3 and 4
17b	For binary outcomes, presentation of both absolute and relative effect sizes is recommended	Not applicable	N/A
Ancillary analyses:			

For peer review only

Section/topic and item No	Standard checklist item	Extension for pilot trials	Page No where item is reported
18	Results of any other analyses performed, including subgroup analyses and adjusted analyses, distinguishing prespecified from exploratory	Results of any other analyses performed that could be used to inform the future definitive trial	N/A
Harms:			
19	All important harms or unintended effects in each group (for specific guidance see CONSORT for harms)		No harms
19a		If relevant, other important unintended consequences	N/A
Discussion			
Limitations:			
20	Trial limitations, addressing sources of potential bias, imprecision, and, if relevant, multiplicity of analyses	Pilot trial limitations, addressing sources of potential bias and remaining uncertainty about feasibility	Pg 11
Generalisability:			
21	Generalisability (external validity, applicability) of the trial findings	Generalisability (applicability) of pilot trial methods and findings to future definitive trial and other studies	Pg 10 and 11
Interpretation:			
22	Interpretation consistent with results, balancing benefits and	Interpretation consistent with pilot trial objectives and findings,	Pg 9, 10 and 11

For peer review only

Section/topic and item No	Standard checklist item	Extension for pilot trials	Page No where item is reported
	harms, and considering other relevant evidence	balancing potential benefits and harms, and considering other relevant evidence	
22a		Implications for progression from pilot to future definitive trial, including any proposed amendments	Pg 10 and 11
Other information			
Registration:			
23	Registration number and name of trial registry	Registration number for pilot trial and name of trial registry	ISRCTN16789770
Protocol:			
24	Where the full trial protocol can be accessed, if available	Where the pilot trial protocol can be accessed, if available	Submitted as supplementary file at BMJ open
Funding:			
25	Sources of funding and other support (such as supply of drugs), role of funders		NIHR HS&DR Programme
26		Ethical approval for the study was granted by the national Social Care Research Ethics Committee 14/IEC08/1018	

For peer review only

BMJ Open

Compassionate care intervention for hospital nursing teams caring for older people: a pilot cluster randomised controlled trial

Journal:	*BMJ Open*
Manuscript ID	bmjopen-2017-018563.R2
Article Type:	Research
Date Submitted by the Author:	06-Nov-2017
Complete List of Authors:	Gould, Lisa; University of Southampton Faculty of Health Sciences, University of Southampton<br>Griffiths, Peter; University of Southampton, Faculty of Health Sciences; NIHR Collaboration for Leadership in Applied Health Research and Care for Wessex<br>Barker, Hannah; University of Southampton Faculty of Health Sciences<br>Libberton, Paula; University of Southampton Faculty of Health Sciences, University of Southampton<br>Mesa-Eguiagaray, Ines; University of Southampton Faculty of Health Sciences, University of Southampton<br>Pickering, Ruth; University of Southampton Faculty of Health Sciences, University of Southampton<br>Shipway, Lisa; University of Southampton Faculty of Health Sciences, University of Southampton<br>Bridges, Jackie; University of Southampton; NIHR Collaboration for Leadership in Applied Health Research and Care for Wessex
**Primary Subject Heading**:	Health services research
Secondary Subject Heading:	Nursing
Keywords:	Compassion, Cluster randomised trial, Hospital, Nursing, Pilot, Older people

SCHOLARONE™
Manuscripts

Compassionate care intervention for hospital nursing teams caring for older people: a pilot cluster randomised controlled trial

Compassionate care intervention for hospital nursing teams caring for older people: a pilot cluster randomised controlled trial

Authors:

Dr Lisa Gould,[1*] Professor Peter Griffiths[1,2] Ms Hannah Barker,[1] Mrs Paula Libberton,[1] Miss Ines Mesa-Eguiagaray,[1] Dr Ruth M.Pickering,[1] Shipway, Lisa, Jane[1], Professor Jackie Bridges,[1,2]

Institutional affiliations of authors:

[1]Faculty of Health Sciences, University of Southampton

[2]NIHR CLAHRC Wessex

Competing interests: none declared

Data can be requested from the corresponding author

[*]**Corresponding author's contact details**: Dr Lisa Gould, University of Southampton, Faculty of Health Sciences, Nightingale Building, University Road, Highfield, Southampton, SO17 1BJ, United Kingdom. L.j.gould@soton.ac.uk

Keywords: Compassion, Nursing, Older people, Cluster randomised trial, Hospital, Pilot

Word count: 3891

Article Summary: Strengths and Limitations of this study

- Findings from this pilot trial make an important contribution to the evidence base on the evaluation of compassionate care interventions, particularly the measurement of patient-based outcomes with older patient groups.
- This study demonstrates that use of experimental method in this field is feasible.
- The study demonstrates where blinding was effective, and where it was more difficult in a pragmatic hospital based study.
- Only six wards were included in this study, meaning the results are not generalizable.
- The study is of insufficient scale to draw meaningful conclusions about CLECC's effectiveness. The findings indicate, however, that more definitive evaluation is merited.

For peer review only

For peer review only - http://bmjopen.bmj.com/site/about/guidelines.xhtml

Compassionate care intervention for hospital nursing teams caring for older people: a pilot cluster randomised controlled trial

Introduction

Healthcare systems internationally are challenged by the provision of optimal care to an aging population.[1] Research into outcomes for older people admitted to hospital is far from encouraging with hospitalised older people at significant risk of functional decline [2] and frail older patients at increased risk of mortality and re-admission.[3] A recent systematic review on outcomes for older people in acute care suggest there is an "urgent need for the development and evaluation of effective interventions…. that optimise the care outcomes of older patients".[4] This review found personalised treatment plans, and clear communication strategies can reduce re-admission and mortality.[4] This study aims to pilot an intervention aimed at improving compassionate hospital care for older people.

Research indicates that the quality of relationships with staff is key to shaping older people's hospital experiences, with older people valuing being seen as people, listened to and involved in treatment.[5] However, evidence from English NHS and international reports [1 6-8] indicates that older people frequently fail to experience positive and caring staff attitudes and behaviours, resulting in a perceived lack of compassion. Expressed simply, compassion is "a deep awareness of the suffering of another coupled with the wish to relieve it".[9] There are four key components to the narrative of nursing compassion.[10] The first focuses on ideas about the *moral attributes* of a 'compassionate' nurse, including wisdom, humanity, love, and empathy. These moral attributes are expressed through a kind of *situational awareness* in which vulnerability and suffering are perceived and acknowledged. These perceptions underpin participation of the nurse in *responsive action* that is aimed at relieving suffering and ensuring dignity, and which involves the nurse in a participatory relationship in which the nurse exercises *relational capacity* through which empathy is experienced and a caring pastoral relationship is constructed.[10,11]

The apparent need to improve compassionate hospital care for older people has led to the development of a number of interventions, but there is a lack of evidence for their efficacy, with utility limited by a seeming reluctance to use rigorous experimental methods for evaluation. A recent systematic review of evidence for compassionate nursing care

For peer review only - http://bmjopen.bmj.com/site/about/guidelines.xhtml

Compassionate care intervention for hospital nursing teams caring for older people: a pilot cluster randomised controlled trial

interventions found that most of the 24 studies identified used uncontrolled before and after designs, with just four using randomised controlled designs.[10] Studies tended to be single-site and small-scale. A wide range of outcome measures were deployed between the studies including staff-based outcomes (e.g. empathy), patient-based outcomes (e.g. mood) and care outcomes (e.g. patient-centeredness), indicating a lack of consensus in the field as to appropriate compassionate care outcomes and how to measure them. While most studies (79%) reported a positive effect in relation to one or more outcomes, higher quality studies were less likely to report positive effects and no interventions were evaluated more than once. Thus the quality of the evidence for effectiveness in this field is predominantly low, hampered by a lack of experimental research of sufficient scale.

Responding to an absence of high quality evidence for the effectiveness of compassionate care interventions for older patients, the study reported here aimed to pilot the use experimental methodology to evaluate a compassionate care intervention targeted at work teams in acute care settings. We aimed to provide an evidence base to guide future trial design and implementation, including feasibility of ward level randomisation, selection of outcome measures including success in blinding, sample size calculation, minimising contamination between experimental and control clusters, and maximising participation of older patients.

Methods

As part of a wider feasibility study, a multi-site pilot cluster randomised controlled trial (CRT) was undertaken with randomisation of staff and patients at ward nursing team level.[12] Medical and surgical wards with high proportion of older patients were eligible. Six wards in two NHS hospital Trusts in England were enrolled and allocated to intervention (n=4) or control (n=2). The number of clusters was determined by funding availability and the plan to run the study in at least two hospital organisations, and at least two ward specialties. Randomisation of clusters was undertaken using the ralloc command in Stata[13] by the team statistician (IM) blinded to hospital and ward information other than ward specialty. Randomisation was stratified by hospital and by ward type: Medicine for Older People

For peer review only - http://bmjopen.bmj.com/site/about/guidelines.xhtml

(MOP) or not MOP. The allocation was communicated to the chief investigator (JB) who oversaw its implementation in practice.

The CLECC intervention is based on workplace learning theory with the ward conceptualised as a learning environment and ward team as a community of practice.[14] It is an educational programme focused on developing manager and team practices at a group level that create an expansive learning environment, theorised to enhance team capacity to provide compassionate care.[15] Expansive (rather than restrictive) environments foster workplace learning and the integration of personal and organisational development.[16-18] The intervention aims to embed ward-based manager and team practices including dialogue, reflective learning and mutual support. Research suggests that embedding such practices leads to a longer-term period of service improvement and sustainable improvements in practice.[19] CLECC training consisted of key activities, such as: monthly ward leader action learning sets; team learning activities, including local team climate analysis and values clarification; peer observations of practice and feedback to team by volunteer team members; team study days focused on team building and understanding patient experiences; mid-shift 5 minute team cluster discussions; and twice weekly team reflective discussions. A Practice Educator led these activities through a four-month implementation period, aiming to develop a team-learning plan that included measures for continuing to support leader and team practices that underpin the delivery of compassionate care beyond the initial programmed activities. Usual practice continued on control wards. Further detail on the theory and development of the CLECC intervention can be found in Bridges and Fuller.[20]

Outcome measures were assessed at baseline (2 months before intervention and prior to randomisation to groups) and follow-up (4 months after completion of CLECC implementation period). Given anticipated patient and staff turnover between assessment periods, follow-up was at cluster level rather than individual participant level, and so recruitment for baseline and follow-up assessment periods was independent. There is no single validated measure for compassionate care, the systematic review cited above identifying 18 different types of outcome measure (a total of 67 individual outcome measures) for compassionate nursing care.[10] The most commonly used nurse-based

For peer review only - http://bmjopen.bmj.com/site/about/guidelines.xhtml

measure identified in the review was empathy, with other measures including compassion, caring and wellbeing, including burnout and stress. Patient-based measures focused on overall satisfaction, quality of life, mood, agitation and wellbeing. Of measures that focused more on care quality, most studies used measures of the quality of interaction between nurses and patients. We chose to assess the performance of three complementary core outcomes: researcher-rated observations of the quality of staff-patient interactions, patient-reported evaluations of emotional care and nurse-reported measures of empathy. Baseline and follow-up data were also gathered on individual and ward team characteristics including patient age, cognitive impairment, ward leadership and staff turnover. We aimed to maximise the participation of older people with cognitive impairment and communication difficulties through recruitment procedures that optimised capacity to make decisions about taking part in the study.[12] Because there is insufficient literature to guide the recruitment of these groups, it was not possible at the outset to predict sample size. Instead, more flexible target recruitment rates were used.

The quality of staff-patient interactions was assessed using the Quality of Interactions Schedule (QuIS), a time sampling tool that measures the volume and quality of interactions through observation.[21] Staff-patient interactions are rated as positive social, positive care, neutral, negative protective or negative restrictive. Earlier piloting work has established its validity and reliability in acute settings.[22]

All adult patients on participating wards were assessed for eligibility to be included in observations. Patients were excluded if they were unable to communicate their choices about taking part in the research and a consultee could not be contacted. We also excluded patients who were unconscious or where there were clinical concerns (critically ill, in receipt of palliative care, high infection risk). The patient sample for observations was determined by randomisation of eligible patients, whereby a random number generator indicated the index patient for approach. Index patients were informed about the planned observations and if they agreed the observation could proceed, other eligible patients in the researcher's field of view were approached for inclusion. If the index patient declined to take part, another index patient was randomly selected, and approached as before. Study records were audited to ensure that allocation determined by randomisation was implemented in

For peer review only - http://bmjopen.bmj.com/site/about/guidelines.xhtml

practice. Staff were informed about observations with the option to withdraw if preferred. All interactions between patients and staff were directly observed by a single researcher for two hours and coded (there were 10 x 2 hour observation sessions per ward per 3 week assessment period). Observation sessions were randomly sampled over three weeks from Monday-Friday, 8.00 a.m.-10.00 p.m., and balanced between wards and time of day. Twelve researchers were trained (4 hours classroom and 6 hours field) to undertake observations.

Patient-reported evaluations of emotional care were measured using the Patient Evaluation of Emotional Care during Hospitalisation (PEECH) survey tool which is validated for use in English hospital settings.[23] Designed to measure patient views on the nature of interpersonal interactions with hospital staff and patient-reported assessment of the extent to which therapeutic emotional care has occurred, the subscales are security, knowledge, personal value and connection. PEECH is sensitive to changes in service quality and in ward environment.[24] All eligible patients on the ward were invited to complete a questionnaire. Patients were excluded if there were clinical concerns or if they lacked capacity to consent. If recruited, patients were offered help by the researcher in completing the questionnaire.

Nurses' self-reported empathy was measured using the Jefferson Scale of Empathy (JSE)(Physician/HP version), a 20 item inventory in a 7-point Likert-type format ranging from Strongly Disagree to Strongly Agree with higher scores reflecting a more empathic orientation.[25] The JSE was developed and validated for use by health care workers, the scale is sensitive to changes in individual empathy over time and context.[26 27] All nursing staff (registered nurses and health care assistants) were invited to complete a questionnaire, based on a staff list supplied by the ward manager. Questionnaires in individually named envelopes were distributed by ward managers and returned via an on-ward postbox.

A number of measures were employed to enable allocation concealment and blinding. Clusters were randomly allocated to group following baseline data collection. At follow-up, researchers conducting observations were blinded to allocation, but researchers gathering questionnaire data were aware of ward allocation. It was not possible to conceal allocation from ward team nursing staff. Patients were not informed of allocation.

For peer review only - http://bmjopen.bmj.com/site/about/guidelines.xhtml

Compassionate care intervention for hospital nursing teams caring for older people: a pilot cluster randomised controlled trial

All analyses were carried out on an intention to treat basis. Descriptive statistics were used to show the proportion of participants that consented to participate in study**.** The proportion of QuIS interactions rated for each of the five categories was analysed and the frequencies of patients with the lowest (most negative) scores for each subscale was calculated. The differences between groups were tested using Chi-square test. A three level mixed-effects logistic regression model was fitted to investigate the effect of the CLECC intervention on the likelihood of a negative interaction. Predictive factors were included as fixed effects and presented as odds ratios (OR) with 95% CI, after adjustment for baseline and ward consecutively. Mean PEECH and JSE scores were calculated by subscale and in total, and differences between groups at follow-up were tested using Mann-Whitney U test. In order to determine the appropriate approach for analysis and the design effect when calculating the required sample in a definitive trial, estimates of intracluster correlation were generated for each outcome measure.

A small patient and public involvement (PPI) group and PPI representatives on the Steering Group oversaw and advised on intervention development, study design, selection of outcome measures and research team training.

Results

Six out of seven nursing ward managers invited to take part agreed to randomisation to either intervention or control. Three wards were recruited in each Trust, and all wards remained in the study until it closed. The wards had between 28 and 32 beds and mean patients stays ranged from six days to 19 days. Data were collected between March 2015 and March 2016. Procedures for allocation concealment and blinding proceeded as planned, with the exception of two researcher observers at follow-up reporting that they learned of ward allocation from ward staff. No staff audited following observations reported that their behaviour had changed because they were being observed. Researcher field notes reflect reports from hospital managers that discussions about CLECC between staff on intervention and control wards had the potential to influence practice on the control wards, but we did not detect evidence of contamination.

For peer review only - http://bmjopen.bmj.com/site/about/guidelines.xhtml

Compassionate care intervention for hospital nursing teams caring for older people: a pilot cluster randomised controlled trial

Participant flow

Figure One shows the flow of clusters and participants through the pilot trial. Randomisation took place after baseline data collection, but results are presented by allocation for baseline and follow-up data to enable comparisons between groups.

FIGURE ONE: CONSORT flow diagram

For staff-patient observations, Figure 1 illustrates the number of approaches rather than individual patients, as some patients were invited more than once to be involved. Recruitment rate for observations at baseline was 97% (152 out of 157 approaches to eligible patients), and at follow-up was 90% (157 out of 175). Recruitment rates were similar between intervention and control wards (96% versus 98% at baseline, 90% versus 88% at follow-up). Twenty-three participants declined to participate for reasons including "not feeling up to it" (17%), or "too unwell" (4%). No specific reason was recorded for 70%. In 17% (63 out of 362 approaches) patients were assessed as not having capacity to make the decision to take part. In 67% (42 out of 63) of these occasions, researchers were able to contact a consultee for advice and in 100% of these cases the consultee advised that the patient should participate. A final 273 patients were observed (133 at baseline and 140 at follow-up). The mean age of patients observed was 82 years (84 years in intervention group and 77 in control) (Table 1). Most patients were female (77%) and 25% had evidence of cognitive impairment, with no significant differences by experimental group. All observation data gathered were included in analysis.

Across both assessment periods, 77% (359 out of 464) of eligible patients agreed to take part in the questionnaire survey. Overall recruitment rates were similar between intervention and control wards (77% versus 78%). Most frequent reasons recorded for patients declining participation in the questionnaire survey were "tired" (40%, n=12) and "questionnaire too difficult" (10%, n=3). The most frequent reasons recorded for excluding

For peer review only - http://bmjopen.bmj.com/site/about/guidelines.xhtml

patients were "not having capacity" (43%, n=48) and "very cognitively impaired" (29%, n=32). Ninety nine percent (354 of 359) of patients who consented returned a completed

Table 1: Patient Characteristics

Variable	
**Observations (n=273)**	
**Missing data=0**	
**Age**	
18-30 years	1 (0%)
31-40 years	2 (1%)
41-50 years	7 (3%)
51-60 years	14 (5%)
61-70 years	14 (5%)
More than 70 years	235 (86%)
**Gender**	
Male	63 (23%)
Female	210 (77%)
**Cognitive impairment**	
Yes	68 (25%)
No	205 (75%)
**Questionnaires (n=321)**	
**Missing data=33**	
**Age**	
18-30 years	4 (1%)
31-40 years	3 (1%)
41-50 years	9 (3%)
51-60 years	15 (5%)
61-70 years	24 (7%)
More than 70 years	266 (83%)
**Gender**	
Male	95 (30%)
Female	226 (70%)
**Cognitive impairment**	
Yes	[n=43]12%
No	[n=315] 88%
Missing	1

For peer review only - http://bmjopen.bmj.com/site/about/guidelines.xhtml

questionnaire, with researchers helping with completion in 68% of cases. Most patients were female (70%), and aged over 70 years (83%). Twelve percent of patient questionnaires were completed by patients with cognitive impairment. Intervention group patients completing questionnaires at baseline included a higher proportion of younger patients (22% aged ≤60 years versus 0%) and of males (43% versus 25%). There were no other notable differences by experimental group (Table 1).

Of 496 questionnaires distributed to nursing staff, 36% (n=178) were completed and returned (37% at baseline, 35% at follow-up). Baseline return rates were lower on intervention wards (31% versus 48%), but at follow-up were more similar between experimental groups (33% versus 39%). Most staff who returned a completed questionnaire were female (87%) and median age group was 26-35 years. Questionnaires were returned by 74 health care assistants (42%), 74 staff nurses (42%), and 18 sisters/charge nurses (10%), (missing data=6%). There were no notable differences in job role by experimental group. All returned questionnaires (91 at baseline and 87 at follow-up) were included in analyses.

Baseline and outcome measures

As planned, 120 hours of observations took place in each assessment period, resulting in data collected on 3109 interactions between staff and patients over 240 hours. On average, each patient had 6 interactions with hospital staff per hour. Most interactions were rated as positive care (59%) and least interactions as negative protective (4%) for each experimental group at both assessment periods (Table 2).

	Baseline (n=1554)		Follow-up (n=1555)	
QuIS rating	CLECC (n=1143)	Control (n=411)	CLECC (n=1119)	Control (n=436)
Positive social	167 (15%)	37 (9%)	243 (22%)	64 (14%)
Positive care	672 (59%)	255 (62%)	632 (57%)	260 (60%)
Neutral	190 (17%)	77 (19%)	151 (14%)	62 (14%)

For peer review only - http://bmjopen.bmj.com/site/about/guidelines.xhtml

Compassionate care intervention for hospital nursing teams caring for older people: a pilot cluster randomised controlled trial

Negative protective	42 (4%)	17 (4%)	36 (3%)	21 (5%)
Negative restrictive	72 (6%)	25 (6%)	57 (5%)	29 (7%)

Table 2 Quality of staff-patient interaction QuIS by experimental group (baseline and follow-up)

At follow-up, there were more total positive (positive social and positive care) and less total negative (negative protective and negative restrictive) scores for intervention wards than control (78% versus 74%, 8% versus 12%). Chi square testing suggested these differences were significant (p=0.017). However, multilevel logistic regression results indicate that once other variables are taken into account, the odds of a negative interaction are not significantly reduced because of the effect of the CLECC intervention (Table 3). Results are in the direction of an effect favourable to CLECC, that is, there were less negative interactions on intervention wards, but this is not a statistically significant difference (adjusted OR 0.30 [95% confidence interval 0.07, 1.32]).

**Variables**	**Model 1 unadjusted OR [95% CI] (n=3,111)**	**Model 2 adjusted OR [95% CI] (n=3,111)**	**Model 3 adjusted OR [95% CI] (n=3,111)**
CLECC effect	0.72 [0.35, 1.51]	0.47 [0.17, 1.29]	0.30 [0.07, 1.32]
Time period (Baseline vs follow-up)		0.56 [0.22, 1.43]	0.38 [0.11, 1.32]
Ward			
A			1.00
B			0.60 [0.20, 1.83]
C			0.80 [0.21, 3.05]
D			0.75 [0.24, 2.35]
E			0.61 [0.19, 1.90]
F			0.23 [0.05, 1.02]
**Variance component estimates (95% CI)**			
Observation session level (n=120)	2.13 [1.25, 3.62]	2.09 [1.23, 3.55]	1.96 [1.14, 3.37]
Patient level (n=273)	0.51 [0.23, 1.13]	0.51 [0.23, 1.13]	0.51 0.23, 1.13]

For peer review only - http://bmjopen.bmj.com/site/about/guidelines.xhtml

Compassionate care intervention for hospital nursing teams caring for older people: a pilot cluster randomised controlled trial

Table 3 QuIS multilevel logistic regression results: odds ratios (OR) of a negative interaction

Table 4 shows the mean patient evaluations of emotional care (PEECH) values by experimental group. Higher scores indicate better patient-reported experiences. Connection subscale scores were consistently lower than on other subscales. Differences between groups at follow-up favor CLECC in total score and three of the four subscales, but these differences were not significant.

PEECH Mean (SD)	Baseline (n=168)		Follow up (n=186)		P value
	CLECC (n=105)	Control (n=63)	CLECC (n=123)	Control (n=63)	
Security (0 to 3)	2.48 (0.55)	2.36 (0.51)	2.48 (0.50)	2.46 (0.48)	0.653
Knowing (0 to 3)	2.18 (0.82)	2.30 (0.72)	2.19 (0.88)	2.26 (0.66)	0.800
Personal value (0 to 3)	2.34 (0.57)	2.35 (0.58)	2.43 (0.57)	2.31 (0.57)	0.071
Connection (0 to 3)	1.68 (0.74)	1.61 (0.84)	1.81 (0.82)	1.71 (0.63)	0.350
Total PEECH score (0 to 66)	49.2 (11.5)	48.4 (12)	50.6 (11.3)	48.5 (9.8)	0.116

Table 4 PEECH mean (SD) scores by experimental group (baseline and follow-up)

Levels of staff self-reported empathy using Jefferson Scale of Empathy varied across individual wards at baseline and at follow-up. There was no significant difference between groups (p=0.800).

At ward level, intracluster correlations (ICCs) for QuIS, PEECH and JSE were low (<0.027). The ICC for QuIS at ward level was higher, although still small (0.071), but high at observation session level (0.411).

Discussion:

This study aimed to deliver a compassionate care intervention in acute care settings, pilot the use of experimental methodology and assess the performance of selected outcome

For peer review only - http://bmjopen.bmj.com/site/about/guidelines.xhtml

measures. We aimed to provide an evidence base to guide future trial design and implementation, including acceptability of ward level randomisation, the feasibility of assessing outcome measures, and other measures of trial implementation such as recruitment and inclusivity, sample size calculation and clustering for future trial, blinding and contamination. The high recruitment rate of ward managers on behalf of their teams and subsequent lack of attrition of any of the ward teams recruited indicate that trial randomisation and the CLECC intervention are acceptable to medical and surgical nursing teams in acute care hospitals. Recruitment processes and methods appeared to be inclusive of all nursing staff levels and of older patients. Observations, in particular, were highly acceptable to patients with an overall recruitment rates of 93%. Questionnaire response rates varied, as discussed below. Our findings suggest that the CLECC intervention may have a favourable effect in reducing negative interactions between staff and patients, and in reducing patients' experiences of lack of emotional connection with staff. However as expected, because of the scale of this pilot, there is no certainty that any apparent positive effects are not produced by chance alone, rather than the impact of the CLECC intervention.

Hospitalised older patients with cognitive impairment are a traditionally hard-to-reach group and even though they appear more prone to negative experiences of hospital care,[28] they are often excluded from research.[5 29 30] It is estimated that up to 25% of beds in acute hospitals are occupied by people with dementia, with the figure likely to be higher on specialist older people's wards.[31 32] While cognitive deficits may limit some people's ability to share their experiences, our study has been successful in devising recruitment and data collection methods that maximise their inclusion. Overall 25% of patients observed in this study had evidence of cognitive impairment, suggesting a sample representative of the wider hospital population. Twelve percent of patient questionnaires returned were completed by patients with cognitive impairment, indicating the questionnaire method was less inclusive than observation methods. Participating in an observation does not require any particular state of health, abilities or performance form the patient in question, whereas participating in a questionnaire about one's care experiences requires a minimum orientation to place, language skills and attention.[30] In addition, using questionnaire

For peer review only - http://bmjopen.bmj.com/site/about/guidelines.xhtml

methods may be psychologically threatening to patients still in receipt of care, regardless of cognitive status.[33]

The validity of observer ratings as accurate representation of patient experiences merits attention. Because main study observation and questionnaire data were gathered from different patient groups, it was not possible to test the validity of observer ratings against patient-reported experience. However, in earlier piloting work we found 79% agreement (weighted kappa 0.40: $P < 0.001$; indicating fair agreement) between patients' and observers' ratings of interaction quality.[22] Our earlier work did not include people with a cognitive impairment and validation of QuIS ratings with this patient group may be a necessary next step in the tool's development. In addition, if the proportion of negative interactions is the primary outcome measure in a future study, understanding which interactions are rated by observers (and, where possible, patients) as negative, and why, is an important next step, as is working with patient representatives to establish their views on the size of a meaningful reduction in negative interactions. Further study can also be used to develop more effective procedures to blind observers from experimental allocation in advance of an experimental study. In addition, the high intrucluster correlation we found at an observation session level merits the exploration of the cause of this variance and the feasibility of different approaches to data collection that reduce its impact, for instance, shorter observation sessions. Our findings echo those of Goldberg et al.[29] that structured non-participant observation appears to be the most promising method to describe the experiences of older people with cognitive impairment in the general hospital setting, and so further evaluation and testing of QuIS across these parameters would be a valuable foundation to its further use as an outcome measure in acute settings.[29]

While the response rate to patient questionnaires was good (77%), of all the patient questionnaires returned, just 12% were completed by patients with cognitive impairment. While questionnaires provide an opportunity for patient to directly rate their care, less successful recruitment of a group known to be vulnerable to more negative experiences in hospital, means that any results may not be a valid representation of this group's experiences. The response rate to nursing questionnaires was low (36%), with some larger scale studies showing response rates of European nurses to be 62%, and US nurses to be

For peer review only - http://bmjopen.bmj.com/site/about/guidelines.xhtml

around 39% .[34] Improving staff survey response rates through further feasibility work would improve confidence that conclusions in empathy levels across staff groups can be drawn with more confidence.

This study was piloted on a small number of wards in two hospitals so the findings are not generalisable. In addition, being observed could, in itself, change staff behaviours, and a common limitation of trials of this kind when it is not possible to conceal allocation from staff, is that bias may influence staff responses to observations and questionnaires. Additionally the finding of possible contamination between wards means that intervention and control conditions should not run in the same organisation over the same time period.

Findings from our wider study, reported elsewhere, that implementation of the CLECC intervention was uneven between wards, difficult to sustain and dependent on organisational support,[35] indicate that, while experimental research in this field is necessary, it will not provide sufficient explanation of results if conducted in isolation. However, the findings reported here represent valuable groundwork to the further development of sound experimental design in a field in which good design and implementation are very much needed.

For peer review only - http://bmjopen.bmj.com/site/about/guidelines.xhtml

Compassionate care intervention for hospital nursing teams caring for older people: a pilot cluster randomised controlled trial

Contributorship statement:

Lisa Gould: implementation, data collection, analysis, interpretation of results, write up of paper.
Jackie Bridges: design, implementation, data collection, analysis, interpretation of results, write up of paper.
Peter Griffiths: design, analysis, interpretation of results, and write up of paper.
Ruth Pickering: design, analysis, interpretation of results, and write up of paper.
Ines Mesa-Eguiagaray: analysis and interpretation of results, write up of paper.
Hannah Barker: implementation, data collection, analysis, interpretation of results, write up of paper.
Lisa Shipway: implementation, data collection, analysis, interpretation of results, write up of paper.
Paula Libberton: implementation, data collection, analysis, interpretation of results, write up of paper.

Acknowledgements
The authors thank all patients, research nurses and staff in the participating hospitals. Without their support, the study would not have been possible. With thanks to our fellow investigators not listed as authors: Rosemary Chable, Emma Munro, Alison Fuller, James Raftery, Avan Aihie Sayer, Greta Westwood, Wendy Wigley and Guiqing Yao.

Funding Acknowledgement:
This project was funded by the National Institute for Health Research [Health Services and Delivery Research programme] (project number 13/07/48)

Department of Health Disclaimer:
The views and opinions expressed therein are those of the authors and do not necessarily reflect those of the Health Services and Delivery Research programme, NIHR, NHS or the Department of Health.

References

1. Christensen K, Doblhammer G, Rau R, et al. Ageing populations: the challenges ahead. *Lancet* 2009;374(9696):1196-208. doi: 10.1016/S0140-6736(09)61460-4
2. Huang HT, Chang CM, Liu LF, et al. Trajectories and predictors of functional decline of hospitalised older patients. *J Clin Nurs* 2013;22(9-10):1322-31. doi: 10.1111/jocn.12055
3. Dent E, Chapman I, Howell S, et al. Frailty and functional decline indices predict poor outcomes in hospitalised older people. *Age Ageing* 2014;43(4):477-84. doi: 10.1093/ageing/aft181
4. Hickman LD, Phillips JL, Newton PJ, et al. Multidisciplinary team interventions to optimise health outcomes for older people in acute care settings: A systematic review. *Arch Gerontol Geriatr* 2015;61(3):322-9. doi: 10.1016/j.archger.2015.06.021
5. Bridges J, Flatley M, Meyer J. Older people's and relatives' experiences in acute care settings: systematic review and synthesis of qualitative studies. *International Journal of Nursing Studies* 2010;47(1):89-107.
6. Francis R. Independent Inquiry into care provided by Mid Staffordshire NHS Foundation Trust January 2005 – March 2009. https://www.gov.uk/government/uploads/system/uploads/attachment_data/file/279109/0375_i.pdf [Accessed 18.02.2017] London: The Stationary Office 2010.
7. Francis R. Report of the Mid Staffordshire NHS Foundation Trust public inquiry. http://webarchive.nationalarchives.gov.uk/20150407084003/http://www.midstaffspublicinquiry.com/report [Accessed 18.02.2017] London, 2013.

For peer review only - http://bmjopen.bmj.com/site/about/guidelines.xhtml

Compassionate care intervention for hospital nursing teams caring for older people: a pilot cluster randomised controlled trial

8. Care Quality Commission. Dignity and nutrition inspection programme: national overview. https://www.cqc.org.uk/sites/default/files/documents/20111007_dignity_and_nutrition_inspection_report_final_update.pdf [Accessed 18.02.2017] Newcastle upon Tyne: Care Quality Commission, 2011.
9. Chochinov H. Dignity and the essence of medicine: the A, B, C, and D of dignity conserving care. *British Medical Journal* 2007;335:184-87.
10. Hartrick G. Relational capacity: the foundation for interpersonal nursing practice. *Journal of Advanced Nursing* 1997;26(3):523-28.
11. Blomberg K, Griffiths P, Wengstrom Y, et al. Interventions for compassionate nursing care: a systematic review. *International Journal of Nursing Studies* 2016;62:137-55.
12. Barker H, Griffiths P, Gould L, et al. Quantity and quality of interaction between staff and older patients in UK hospital wards: a descriptive study. *International Journal of Nursing Studies* 2016;62:100-07.
13. StataCorp. Stata Statistical Software: Release 12. College Station, TX: StataCorp LP, 2011.
14. Fuller A, Unwin L. Expansive learning environments: integrating organizational and personal development. In: Rainbird H, Fuller A, Munro A, eds. Workplace learning in context. London: Routledge 2004:126-44.
15. Bridges J, Fuller A. Creating Learning Environments for Compassionate Care (CLECC): a programme to promote compassionate care by health and social care teams. *International Journal of Older People Nursing* 2015;10(1):48-58.
16. Fuller A. Theories of learning and communities of practice. In: Hughes J, Jewson N, Unwin L, eds. Learning in communities of practice. London: Routledge 2007.
17. Fuller A, Unwin L. Expansive learning environments: integrating organisational and personal development. In: Rainbird H, Fuller A, Munro A, eds. Workplace learning in context. London: Routledge 2004:126-44.
18. Wenger E. Communities of practice: learning, meaning and identity. Cambridge: Cambridge University Press 1998.
19. May C, Finch T, Mair F, et al. Understanding the implementation of complex interventions in health care: the normalization process model. *BMC Health Services Research* 2007;7(1):148.
20. Bridges J, Fuller A. Creating learning environments for compassionate care: a programme to promote compassionate care by health and social care teams. *Int J Older People Nurs* 2015;10(1):48-58. doi: 10.1111/opn.12055
21. Dean R, Proudfoot R, Lindesay J. The quality of interactions schedule(QUIS): development, reliability and use in the evaluation of two domus units. *International Journal of Geriatric Psychiatry* 1993;8:819-26.
22. McLean C, Griffiths P, Mesa-Eguiagaray I, et al. Reliability, feasibility, and validity of the quality of interactions schedule (QuIS) in acute hospital care: an observational study. *BMC Health Serv Res* 2017;17(1):380. doi: 10.1186/s12913-017-2312-2
23. Murrells T, Robert G, Adams M, et al. Measuring relational aspects of hospital care in England with the 'Patient Evaluation of Emotional Care during Hospitalisation'(PEECH) survey questionnaire. *BMJ Open* 2013;3(1)
24. Williams A, Dawson S, Kristjanson L. Translating theory into practice: using action research to introduce a coordinated approach to emotional care. *Patient Education and Counseling* 2008;73(1):82-90.
25. Hojat M, Mangione S, Nasca TJ, et al. The Jefferson Scale of Physician Empathy: development and preliminary psychometric data. *Educational and Psychological Measurement* 2001;61(2):349-65.
26. Hojat M, Vergare MJ, Maxwell K, et al. The devil is in the third year: a longitudinal study of erosion of empathy in medical school. *Academic Medicine* 2009;84(9):1182-91.

For peer review only - http://bmjopen.bmj.com/site/about/guidelines.xhtml

Compassionate care intervention for hospital nursing teams caring for older people: a pilot cluster randomised controlled trial

27. Hojat M, Mangione S, Nasca TJ, et al. An empirical study of decline in empathy in medical school. *Medical Education* 2004;38(9):934-41.
28. Hartley P, Gibbins N, Saunders A, et al. The association between cognitive impairment and functional outcome in hospitalised older patients: a systematic review and meta-analysis. *Age Ageing* 2017 doi: 10.1093/ageing/afx007
29. Goldberg SE, Harwood RH. Experience of general hospital care in older patients with cognitive impairment: are we measuring the most vulnerable patients' experience? *BMJ Quality & Safety* 2013;22(12):977-80.
30. van Baalen A, Vingerhoets AJJM, Sixma HJ, et al. How to evaluate quality of care from the perspective of people with dementia: an overview of the literature. *Dementia* 2010;10(1):112-37.
31. Alzheimers Society. Counting the cost: Caring for people with dementia on hospital wards. 2009
32. Mukadam N, Sampson EL. A systematic review of the prevalence, associations and outcomes of dementia in older general hospital inpatients. *International Psychogeriatrics* 2011;23(3):344-55.
33 La Monica EL, Wolf RM, Madea AR, et al. Empathy and nursing care outcomes. *Scholarly Inquiry for Nursing Practice* 1987;1(3):197-213.
34. Aiken LH, Sermeus W, Van den Heede K, et al. Patient safety, satisfaction, and quality of hospital care: cross sectional surveys of nurses and patients in 12 countries in Europe and the United States. *BMJ: British Medical Journal* 2012;344
35. Bridges J, May C, Fuller A, et al. Optimising impact and sustainability: a qualitative process evaluation of a complex intervention targeted at compassionate care. *BMJ Qual Saf* 2017 doi: 10.1136/bmjqs-2017-006702

For peer review only

For peer review only - http://bmjopen.bmj.com/site/about/guidelines.xhtml

CONSORT Participant Flow Diagram for Pilot Cluster RCT

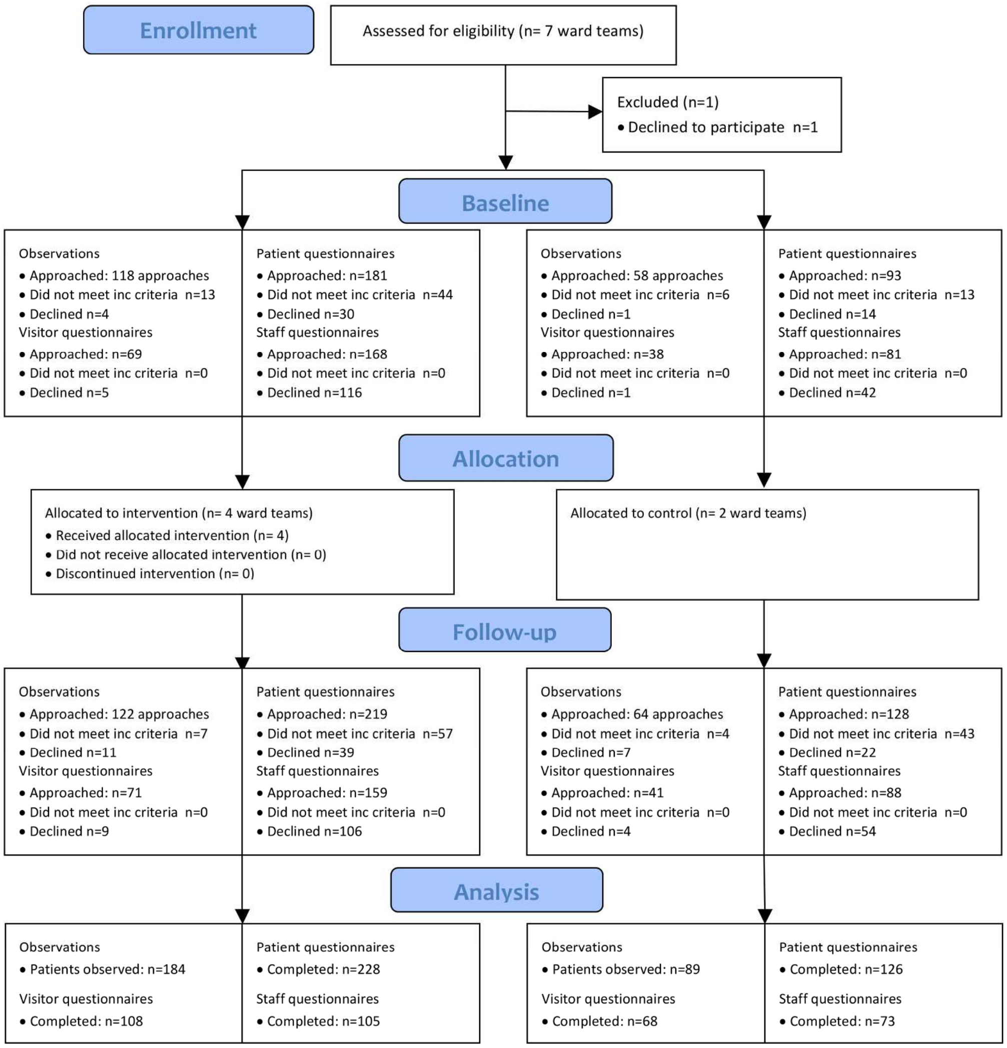

187x222mm (300 x 300 DPI)

Table 2
CONSORT checklist of information to include when reporting a pilot trial

Section/topic and item No	Standard checklist item	Extension for pilot trials	Page No where item is reported
Title and abstract			
1a	Identification as a randomised trial in the title	Identification as a pilot or feasibility randomised trial in the title	Title (not allocated page number yet)
1b	Structured summary of trial design, methods, results, and conclusions (for specific guidance see CONSORT for abstracts)	Structured summary of pilot trial design, methods, results, and conclusions (for specific guidance see CONSORT abstract extension for pilot trials)	Abstract (not allocated page number yet)
Introduction			
Background and objectives:			
2a	Scientific background and explanation of rationale	Scientific background and explanation of rationale for future definitive trial, and reasons for randomised pilot trial	Pg 1-2
2b	Specific objectives or hypotheses	Specific objectives or research questions for pilot trial	Pg 2
Methods			
Trial design:			
3a	Description of trial design (such as parallel, factorial) including allocation ratio	Description of pilot trial design (such as parallel, factorial) including allocation ratio	Pg 2 and 3
3b	Important changes to methods after trial commencement (such as eligibility criteria), with	Important changes to methods after pilot trial commencement (such as eligibility criteria), with reasons	N/A, no changes made

For peer review only

Section/topic and item No	Standard checklist item	Extension for pilot trials	Page No where item is reported
	reasons		
Participants:			
4a	Eligibility criteria for participants		Pg 4 and 5
4b	Settings and locations where the data were collected		Pg 2
4c		How participants were identified and consented	Pg 3, 4 and 5
Interventions:			
5	The interventions for each group with sufficient details to allow replication, including how and when they were actually administered		Pg 3
Outcomes:			
6a	Completely defined prespecified primary and secondary outcome measures, including how and when they were assessed	Completely defined prespecified assessments or measurements to address each pilot trial objective specified in 2b, including how and when they were assessed	Pg 3, 4 and 5
6b	Any changes to trial outcomes after the trial commenced, with reasons	Any changes to pilot trial assessments or measurements after the pilot trial commenced, with reasons	N/A no changes
6c		If applicable, prespecified criteria used to judge whether, or how, to proceed with future definitive trial	

For peer review only

Section/topic and item No	Standard checklist item	Extension for pilot trials	Page No where item is reported
Sample size:			
7a	How sample size was determined	Rationale for numbers in the pilot trial	Pg 4
7b	When applicable, explanation of any interim analyses and stopping guidelines		N/A
Randomisation:			
Sequence generation:			
8a	Method used to generate the random allocation sequence		Pg 2 and 3 (ward randomisation) and 4 (observation randomisation)
8b	Type of randomisation; details of any restriction (such as blocking and block size)	Type of randomisation(s); details of any restriction (such as blocking and block size)	Pg 2 and 3, and 4
Allocation concealment mechanism:			
9	Mechanism used to implement the random allocation sequence (such as sequentially numbered containers), describing any steps taken to conceal the sequence until interventions were assigned		Pg 2 and 3,4 and 5
Implementation:			
10	Who generated the random allocation sequence, enrolled participants, and assigned participants to interventions		Pg 2, 3, 4 and 5

For peer review only

Section/topic and item No	Standard checklist item	Extension for pilot trials	Page No where item is reported
Blinding:			
11a	If done, who was blinded after assignment to interventions (eg, participants, care providers, those assessing outcomes) and how		Pg 5
11b	If relevant, description of the similarity of interventions		N/A
Analytical methods:			
12a	Statistical methods used to compare groups for primary and secondary outcomes	Methods used to address each pilot trial objective whether qualitative or quantitative	6, 7, 8 and 9
12b	Methods for additional analyses, such as subgroup analyses and adjusted analyses	Not applicable	N/A
Results			
Participant flow (a diagram is strongly recommended):			
13a	For each group, the numbers of participants who were randomly assigned, received intended treatment, and were analysed for the primary outcome	For each group, the numbers of participants who were approached and/or assessed for eligibility, randomly assigned, received intended treatment, and were assessed for each objective	Figure 1, and pg 6, 7 and 8
13b	For each group, losses and exclusions after randomisation, together with reasons		Figure 1, and pg 6, 7 and 8

For peer review only

Section/topic and item No	Standard checklist item	Extension for pilot trials	Page No where item is reported
Recruitment:			
14a	Dates defining the periods of recruitment and follow-up		Pg 6
14b	Why the trial ended or was stopped	Why the pilot trial ended or was stopped	N/A
Baseline data:			
15	A table showing baseline demographic and clinical characteristics for each group		Table 1
Numbers analysed:			
16	For each group, number of participants (denominator) included in each analysis and whether the analysis was by original assigned groups	For each objective, number of participants (denominator) included in each analysis. If relevant, these numbers should be by randomised group	Figure 1, pg 6, 7 and 8
Outcomes and estimation:			
17a	For each primary and secondary outcome, results for each group, and the estimated effect size and its precision (such as 95% confidence interval)	For each objective, results including expressions of uncertainty (such as 95% confidence interval) for any estimates. If relevant, these results should be by randomised group	Pg 8 and 9. Tables 2, 3 and 4
17b	For binary outcomes, presentation of both absolute and relative effect sizes is recommended	Not applicable	N/A
Ancillary analyses:			

For peer review only

Section/topic and item No	Standard checklist item	Extension for pilot trials	Page No where item is reported
18	Results of any other analyses performed, including subgroup analyses and adjusted analyses, distinguishing prespecified from exploratory	Results of any other analyses performed that could be used to inform the future definitive trial	N/A
Harms:			
19	All important harms or unintended effects in each group (for specific guidance see CONSORT for harms)		No harms
19a		If relevant, other important unintended consequences	N/A
Discussion			
Limitations:			
20	Trial limitations, addressing sources of potential bias, imprecision, and, if relevant, multiplicity of analyses	Pilot trial limitations, addressing sources of potential bias and remaining uncertainty about feasibility	Pg 11
Generalisability:			
21	Generalisability (external validity, applicability) of the trial findings	Generalisability (applicability) of pilot trial methods and findings to future definitive trial and other studies	Pg 10 and 11
Interpretation:			
22	Interpretation consistent with results, balancing benefits and	Interpretation consistent with pilot trial objectives and findings,	Pg 9, 10 and 11

For peer review only

Section/topic and item No	Standard checklist item	Extension for pilot trials	Page No where item is reported
	harms, and considering other relevant evidence	balancing potential benefits and harms, and considering other relevant evidence	
22a		Implications for progression from pilot to future definitive trial, including any proposed amendments	Pg 10 and 11
Other information			
Registration:			
23	Registration number and name of trial registry	Registration number for pilot trial and name of trial registry	ISRCTN16789770
Protocol:			
24	Where the full trial protocol can be accessed, if available	Where the pilot trial protocol can be accessed, if available	Submitted as supplementary file at BMJ open
Funding:			
25	Sources of funding and other support (such as supply of drugs), role of funders		NIHR HS&DR Programme
26		Ethical approval for the study was granted by the national Social Care Research Ethics Committee 14/IEC08/1018	

For peer review only

BMJ Open

Compassionate care intervention for hospital nursing teams caring for older people: a pilot cluster randomised controlled trial

Journal:	*BMJ Open*
Manuscript ID	bmjopen-2017-018563.R3
Article Type:	Research
Date Submitted by the Author:	16-Nov-2017
Complete List of Authors:	Gould, Lisa; University of Southampton Faculty of Health Sciences, University of Southampton<br>Griffiths, Peter; University of Southampton, Faculty of Health Sciences; NIHR Collaboration for Leadership in Applied Health Research and Care for Wessex<br>Barker, Hannah; University of Southampton Faculty of Health Sciences<br>Libberton, Paula; University of Southampton Faculty of Health Sciences, University of Southampton<br>Mesa-Eguiagaray, Ines; University of Southampton Faculty of Health Sciences, University of Southampton<br>Pickering, Ruth; University of Southampton Faculty of Health Sciences, University of Southampton<br>Shipway, Lisa; University of Southampton Faculty of Health Sciences, University of Southampton<br>Bridges, Jackie; University of Southampton; NIHR Collaboration for Leadership in Applied Health Research and Care for Wessex
**Primary Subject Heading**:	Health services research
Secondary Subject Heading:	Nursing
Keywords:	Compassion, Cluster randomised trial, Hospital, Nursing, Pilot, Older people

SCHOLARONE™ Manuscripts

Compassionate care intervention for hospital nursing teams caring for older people: a pilot cluster randomised controlled trial

Dr Lisa Gould,[1*] Professor Peter Griffiths[1,2] Ms Hannah Barker,[1] Mrs Paula Libberton,[1] Miss Ines Mesa-Eguiagaray,[1] Dr Ruth M.Pickering,[1] Shipway, Lisa, Jane[1], Professor Jackie Bridges,[1,2]

[1]Faculty of Health Sciences, University of Southampton

[2]NIHR CLAHRC Wessex

Competing interests: none declared

Data can be requested from the corresponding author

[*]**Corresponding author's contact details**: Dr Lisa Gould, University of Southampton, Faculty of Health Sciences, Nightingale Building, University Road, Highfield, Southampton, SO17 1BJ, United Kingdom. L.j.gould@soton.ac.uk

Abstract

OBJECTIVE: Compassionate care continues to be a focus for national and international attention, but the existing evidence base lacks the experimental methodology necessary to guide the selection of effective interventions for practice. This study aimed to evaluate the Creating Learning Environments for Compassionate Care (CLECC) intervention in improving compassionate care.

SETTING: Ward nursing teams (clusters) in two English NHS hospitals randomised to intervention (n=4) or control (n=2). Intervention wards comprised two medicines for older people (MOP) wards and two medical/surgical wards. Control wards were both MOP's.

PARTICIPANTS: Data collected from 627 patients and 178 staff. Exclusion criteria: Reverse barrier nursed, critically ill, palliative or non-English speaking. All other patients and all nursing staff and HCAs were invited to participant, agency and bank staff were excluded.

INTERVENTION: CLECC, a workplace intervention focused on developing sustainable leadership and work-team practices to support the delivery of compassionate care. Control: no educational activity.

PRIMARY AND SECONDARY OUTCOME MEASURES: Primary- Quality of Interaction Schedule (QuIS) for observed staff-patient interactions. Secondary- patient-reported evaluations of emotional care in hospital (PEECH); nurse-reported empathy (Jefferson Scale of Empathy).

RESULTS: Trial proceeded as per protocol, randomisation was acceptable. Some but not all blinding strategies were successful. QuIS observations achieved 93% recruitment rate with 25% of patient sample cognitively impaired. At follow-up there were more total positive (78% versus 74%) and less total negative (8% versus 11%) QuIS ratings for intervention wards versus control wards. Sixty-three percent of intervention ward patients scored lowest (i.e. more negative) scores on PEECH connection subscale, versus 79% of control. This was not a statistically significant difference. No statistically significant differences in nursing empathy were observed.

For peer review only - http://bmjopen.bmj.com/site/about/guidelines.xhtml

CONCLUSIONS: Use of experimental methods is feasible. The use of structured observation of staff-patient interaction quality is a promising outcome measure inclusive of hard to reach groups.

Trial registration: ISRCTN16789770

Strengths and limitations of this study

- Findings from this pilot trial make an important contribution to the evidence base on the evaluation of compassionate care interventions, particularly the measurement of patient-based outcomes with older patient groups.
- This study demonstrates that use of experimental method in this field is feasible.
- The study demonstrates where blinding was effective, and where it was more difficult in a pragmatic hospital based study.
- Only six wards were included in this study, meaning the results are not generalizable.
- The study is of insufficient scale to draw meaningful conclusions about CLECC's effectiveness. The findings indicate, however, that more definitive evaluation is merited.

Keywords: Compassion, Nursing, Older people, Cluster randomised trial, Hospital, Pilot

Word count: 3891

For peer review only - http://bmjopen.bmj.com/site/about/guidelines.xhtml

Compassionate care intervention for hospital nursing teams caring for older people: a pilot cluster randomised controlled trial

Healthcare systems internationally are challenged by the provision of optimal care to an aging population.[1] Research into outcomes for older people admitted to hospital is far from encouraging with hospitalised older people at significant risk of functional decline [2] and frail older patients at increased risk of mortality and re-admission.[3] A recent systematic review on outcomes for older people in acute care suggest there is an "urgent need for the development and evaluation of effective interventions…. that optimise the care outcomes of older patients".[4] This review found personalised treatment plans, and clear communication strategies can reduce re-admission and mortality.[4] This study aims to pilot an intervention aimed at improving compassionate hospital care for older people.

Research indicates that the quality of relationships with staff is key to shaping older people's hospital experiences, with older people valuing being seen as people, listened to and involved in treatment.[5] However, evidence from English NHS and international reports [1 6-8] indicates that older people frequently fail to experience positive and caring staff attitudes and behaviours, resulting in a perceived lack of compassion. Expressed simply, compassion is "a deep awareness of the suffering of another coupled with the wish to relieve it".[9] There are four key components to the narrative of nursing compassion.[10] The first focuses on ideas about the *moral attributes* of a 'compassionate' nurse, including wisdom, humanity, love, and empathy. These moral attributes are expressed through a kind of *situational awareness* in which vulnerability and suffering are perceived and acknowledged. These perceptions underpin participation of the nurse in *responsive action* that is aimed at relieving suffering and ensuring dignity, and which involves the nurse in a participatory relationship in which the nurse exercises *relational capacity* through which empathy is experienced and a caring pastoral relationship is constructed.[10,11]

The apparent need to improve compassionate hospital care for older people has led to the development of a number of interventions, but there is a lack of evidence for their efficacy, with utility limited by a seeming reluctance to use rigorous experimental methods for evaluation. A recent systematic review of evidence for compassionate nursing care interventions found that most of the 24 studies identified used uncontrolled before and

For peer review only - http://bmjopen.bmj.com/site/about/guidelines.xhtml

after designs, with just four using randomised controlled designs.[10] Studies tended to be single-site and small-scale. A wide range of outcome measures were deployed between the studies including staff-based outcomes (e.g. empathy), patient-based outcomes (e.g. mood) and care outcomes (e.g. patient-centeredness), indicating a lack of consensus in the field as to appropriate compassionate care outcomes and how to measure them. While most studies (79%) reported a positive effect in relation to one or more outcomes, higher quality studies were less likely to report positive effects and no interventions were evaluated more than once. Thus the quality of the evidence for effectiveness in this field is predominantly low, hampered by a lack of experimental research of sufficient scale.

Responding to an absence of high quality evidence for the effectiveness of compassionate care interventions for older patients, the study reported here aimed to pilot the use experimental methodology to evaluate a compassionate care intervention targeted at work teams in acute care settings. We aimed to provide an evidence base to guide future trial design and implementation, including feasibility of ward level randomisation, selection of outcome measures including success in blinding, sample size calculation, minimising contamination between experimental and control clusters, and maximising participation of older patients.

Methods

As part of a wider feasibility study, a multi-site pilot cluster randomised controlled trial (CRT) was undertaken with randomisation of staff and patients at ward nursing team level.[12] Medical and surgical wards with high proportion of older patients were eligible. Six wards in two NHS hospital Trusts in England were enrolled and allocated to intervention (n=4) or control (n=2). The number of clusters was determined by funding availability and the plan to run the study in at least two hospital organisations, and at least two ward specialties. Randomisation of clusters was undertaken using the ralloc command in Stata[13] by the team statistician (IM) blinded to hospital and ward information other than ward specialty. Randomisation was stratified by hospital and by ward type: Medicine for Older People (MOP) or not MOP. The allocation was communicated to the chief investigator (JB) who oversaw its implementation in practice.

For peer review only - http://bmjopen.bmj.com/site/about/guidelines.xhtml

The CLECC intervention is based on workplace learning theory with the ward conceptualised as a learning environment and ward team as a community of practice.[14] It is an educational programme focused on developing manager and team practices at a group level that create an expansive learning environment, theorised to enhance team capacity to provide compassionate care.[15] Expansive (rather than restrictive) environments foster workplace learning and the integration of personal and organisational development.[16-18] The intervention aims to embed ward-based manager and team practices including dialogue, reflective learning and mutual support. Research suggests that embedding such practices leads to a longer-term period of service improvement and sustainable improvements in practice.[19] CLECC training consisted of key activities, such as: monthly ward leader action learning sets; team learning activities, including local team climate analysis and values clarification; peer observations of practice and feedback to team by volunteer team members; team study days focused on team building and understanding patient experiences; mid-shift 5 minute team cluster discussions; and twice weekly team reflective discussions. A Practice Educator led these activities through a four-month implementation period, aiming to develop a team-learning plan that included measures for continuing to support leader and team practices that underpin the delivery of compassionate care beyond the initial programmed activities. Usual practice continued on control wards. Further detail on the theory and development of the CLECC intervention can be found in Bridges and Fuller.[20]

Outcome measures were assessed at baseline (2 months before intervention and prior to randomisation to groups) and follow-up (4 months after completion of CLECC implementation period). Given anticipated patient and staff turnover between assessment periods, follow-up was at cluster level rather than individual participant level, and so recruitment for baseline and follow-up assessment periods was independent. There is no single validated measure for compassionate care, the systematic review cited above identifying 18 different types of outcome measure (a total of 67 individual outcome measures) for compassionate nursing care.[10] The most commonly used nurse-based measure identified in the review was empathy, with other measures including compassion, caring and wellbeing, including burnout and stress. Patient-based measures focused on overall satisfaction, quality of life, mood, agitation and wellbeing. Of measures that focused

For peer review only - http://bmjopen.bmj.com/site/about/guidelines.xhtml

more on care quality, most studies used measures of the quality of interaction between nurses and patients. We chose to assess the performance of three complementary core outcomes: researcher-rated observations of the quality of staff-patient interactions, patient-reported evaluations of emotional care and nurse-reported measures of empathy. Baseline and follow-up data were also gathered on individual and ward team characteristics including patient age, cognitive impairment, ward leadership and staff turnover. We aimed to maximise the participation of older people with cognitive impairment and communication difficulties through recruitment procedures that optimised capacity to make decisions about taking part in the study.[12] Because there is insufficient literature to guide the recruitment of these groups, it was not possible at the outset to predict sample size. Instead, more flexible target recruitment rates were used.

The quality of staff-patient interactions was assessed using the Quality of Interactions Schedule (QuIS), a time sampling tool that measures the volume and quality of interactions through observation.[21] Staff-patient interactions are rated as positive social, positive care, neutral, negative protective or negative restrictive. Earlier piloting work has established its validity and reliability in acute settings.[22]

All adult patients on participating wards were assessed for eligibility to be included in observations. Patients were excluded if they were unable to communicate their choices about taking part in the research and a consultee could not be contacted. We also excluded patients who were unconscious or where there were clinical concerns (critically ill, in receipt of palliative care, high infection risk). The patient sample for observations was determined by randomisation of eligible patients, whereby a random number generator indicated the index patient for approach. Index patients were informed about the planned observations and if they agreed the observation could proceed, other eligible patients in the researcher's field of view were approached for inclusion. If the index patient declined to take part, another index patient was randomly selected, and approached as before. Study records were audited to ensure that allocation determined by randomisation was implemented in practice. Staff were informed about observations with the option to withdraw if preferred. All interactions between patients and staff were directly observed by a single researcher for two hours and coded (there were 10 x 2 hour observation sessions per ward per 3 week assessment period). Observation sessions were randomly sampled over three weeks from

For peer review only - http://bmjopen.bmj.com/site/about/guidelines.xhtml

Monday-Friday, 8.00 a.m.-10.00 p.m., and balanced between wards and time of day. Twelve researchers were trained (4 hours classroom and 6 hours field) to undertake observations.

Patient-reported evaluations of emotional care were measured using the Patient Evaluation of Emotional Care during Hospitalisation (PEECH) survey tool which is validated for use in English hospital settings.[23] Designed to measure patient views on the nature of interpersonal interactions with hospital staff and patient-reported assessment of the extent to which therapeutic emotional care has occurred, the subscales are security, knowledge, personal value and connection. PEECH is sensitive to changes in service quality and in ward environment.[24] All eligible patients on the ward were invited to complete a questionnaire. Patients were excluded if there were clinical concerns or if they lacked capacity to consent. If recruited, patients were offered help by the researcher in completing the questionnaire.

Nurses' self-reported empathy was measured using the Jefferson Scale of Empathy (JSE)(Physician/HP version), a 20 item inventory in a 7-point Likert-type format ranging from Strongly Disagree to Strongly Agree with higher scores reflecting a more empathic orientation.[25] The JSE was developed and validated for use by health care workers, the scale is sensitive to changes in individual empathy over time and context.[26 27] All nursing staff (registered nurses and health care assistants) were invited to complete a questionnaire, based on a staff list supplied by the ward manager. Questionnaires in individually named envelopes were distributed by ward managers and returned via an on-ward postbox.

A number of measures were employed to enable allocation concealment and blinding. Clusters were randomly allocated to group following baseline data collection. At follow-up, researchers conducting observations were blinded to allocation, but researchers gathering questionnaire data were aware of ward allocation. It was not possible to conceal allocation from ward team nursing staff. Patients were not informed of allocation.

All analyses were carried out on an intention to treat basis. Descriptive statistics were used to show the proportion of participants that consented to participate in study. The proportion of QuIS interactions rated for each of the five categories was analysed and the frequencies of patients with the lowest (most negative) scores for each subscale was

For peer review only - http://bmjopen.bmj.com/site/about/guidelines.xhtml

calculated. The differences between groups were tested using Chi-square test. A three level mixed-effects logistic regression model was fitted to investigate the effect of the CLECC intervention on the likelihood of a negative interaction. Predictive factors were included as fixed effects and presented as odds ratios (OR) with 95% CI, after adjustment for baseline and ward consecutively. Mean PEECH and JSE scores were calculated by subscale and in total, and differences between groups at follow-up were tested using Mann-Whitney U test. In order to determine the appropriate approach for analysis and the design effect when calculating the required sample in a definitive trial, estimates of intracluster correlation were generated for each outcome measure.

A small patient and public involvement (PPI) group and PPI representatives on the Steering Group oversaw and advised on intervention development, study design, selection of outcome measures and research team training.

Results

Six out of seven nursing ward managers invited to take part agreed to randomisation to either intervention or control. Three wards were recruited in each Trust, and all wards remained in the study until it closed. The wards had between 28 and 32 beds and mean patients stays ranged from six days to 19 days. Data were collected between March 2015 and March 2016. Procedures for allocation concealment and blinding proceeded as planned, with the exception of two researcher observers at follow-up reporting that they learned of ward allocation from ward staff. No staff audited following observations reported that their behaviour had changed because they were being observed. Researcher field notes reflect reports from hospital managers that discussions about CLECC between staff on intervention and control wards had the potential to influence practice on the control wards, but we did not detect evidence of contamination.

Participant flow

Figure One shows the flow of clusters and participants through the pilot trial. Randomisation took place after baseline data collection, but results are presented by allocation for baseline and follow-up data to enable comparisons between groups.

FIGURE ONE: CONSORT flow diagram

For peer review only - http://bmjopen.bmj.com/site/about/guidelines.xhtml

For staff-patient observations, Figure 1 illustrates the number of approaches rather than individual patients, as some patients were invited more than once to be involved. Recruitment rate for observations at baseline was 97% (152 out of 157 approaches to eligible patients), and at follow-up was 90% (157 out of 175). Recruitment rates were similar between intervention and control wards (96% versus 98% at baseline, 90% versus 88% at follow-up). Twenty-three participants declined to participate for reasons including “not feeling up to it” (17%), or “too unwell” (4%). No specific reason was recorded for 70%. In 17% (63 out of 362 approaches) patients were assessed as not having capacity to make the decision to take part. In 67% (42 out of 63) of these occasions, researchers were able to contact a consultee for advice and in 100% of these cases the consultee advised that the patient should participate. A final 273 patients were observed (133 at baseline and 140 at follow-up). The mean age of patients observed was 82 years (84 years in intervention group and 77 in control) (Table 1). Most patients were female (77%) and 25% had evidence of cognitive impairment, with no significant differences by experimental group. All observation data gathered were included in analysis.

Across both assessment periods, 77% (359 out of 464) of eligible patients agreed to take part in the questionnaire survey. Overall recruitment rates were similar between intervention and control wards (77% versus 78%). Most frequent reasons recorded for patients declining participation in the questionnaire survey were “tired” (40%, n=12) and “questionnaire too difficult” (10%, n=3). The most frequent reasons recorded for excluding patients were “not having capacity” (43%, n=48) and “very cognitively impaired” (29%, n=32). Ninety nine percent (354 of 359) of patients who consented returned a completed questionnaire, with researchers helping with completion in 68% of cases. Most patients were female (70%), and aged over 70 years (83%). Twelve percent of patient questionnaires were completed by patients with cognitive impairment. Intervention group patients completing questionnaires at baseline included a higher proportion of younger patients (22% aged ≤60 years versus 0%) and of males (43% versus 25%). There were no other notable differences by experimental group (Table 1).

For peer review only - http://bmjopen.bmj.com/site/about/guidelines.xhtml

Table 1: Patient Characteristics

Variable	
**Observations (n=273)**	
**Missing data=0**	
**Age**	
18-30 years	1 (0%)
31-40 years	2 (1%)
41-50 years	7 (3%)
51-60 years	14 (5%)
61-70 years	14 (5%)
More than 70 years	235 (86%)
**Gender**	
Male	63 (23%)
Female	210 (77%)
**Cognitive impairment**	
Yes	68 (25%)
No	205 (75%)
**Questionnaires (n=321)**	
**Missing data=33**	
**Age**	
18-30 years	4 (1%)
31-40 years	3 (1%)
41-50 years	9 (3%)
51-60 years	15 (5%)
61-70 years	24 (7%)
More than 70 years	266 (83%)
**Gender**	
Male	95 (30%)
Female	226 (70%)
**Cognitive impairment**	
Yes	[n=43]12%
No	[n=315] 88%
Missing	1

Of 496 questionnaires distributed to nursing staff, 36% (n=178) were completed and returned (37% at baseline, 35% at follow-up). Baseline return rates were lower on intervention wards (31% versus 48%), but at follow-up were more similar between experimental groups (33% versus 39%). Most staff who returned a completed questionnaire were female (87%) and median age group was 26-35 years. Questionnaires were returned by 74 health care assistants (42%), 74 staff nurses (42%), and 18 sisters/charge nurses (10%), (missing data=6%). There were no notable differences in job role by experimental group. All returned questionnaires (91 at baseline and 87 at follow-up) were included in analyses.

For peer review only - http://bmjopen.bmj.com/site/about/guidelines.xhtml

Baseline and outcome measures

As planned, 120 hours of observations took place in each assessment period, resulting in data collected on 3109 interactions between staff and patients over 240 hours. On average, each patient had 6 interactions with hospital staff per hour. Most interactions were rated as positive care (59%) and least interactions as negative protective (4%) for each experimental group at both assessment periods (Table 2).

QuIS rating	Baseline (n=1554)		Follow-up (n=1555)	
	CLECC (n=1143)	Control (n=411)	CLECC (n=1119)	Control (n=436)
Positive social	167 (15%)	37 (9%)	243 (22%)	64 (14%)
Positive care	672 (59%)	255 (62%)	632 (57%)	260 (60%)
Neutral	190 (17%)	77 (19%)	151 (14%)	62 (14%)
Negative protective	42 (4%)	17 (4%)	36 (3%)	21 (5%)
Negative restrictive	72 (6%)	25 (6%)	57 (5%)	29 (7%)

Table 2 Quality of staff-patient interaction QuIS by experimental group (baseline and follow-up)

At follow-up, there were more total positive (positive social and positive care) and less total negative (negative protective and negative restrictive) scores for intervention wards than control (78% versus 74%, 8% versus 12%). Chi square testing suggested these differences were significant (p=0.017). However, multilevel logistic regression results indicate that once other variables are taken into account, the odds of a negative interaction are not significantly reduced because of the effect of the CLECC intervention (Table 3). Results are in the direction of an effect favourable to CLECC, that is, there were less negative interactions on intervention wards, but this is not a statistically significant difference (adjusted OR 0.30 [95% confidence interval 0.07, 1.32]).

For peer review only - http://bmjopen.bmj.com/site/about/guidelines.xhtml

Variables	Model 1 unadjusted OR [95% CI] (n=3,111)	Model 2 adjusted OR [95% CI] (n=3,111)	Model 3 adjusted OR [95% CI] (n=3,111)
CLECC effect	0.72 [0.35, 1.51]	0.47 [0.17, 1.29]	0.30 [0.07, 1.32]
Time period (Baseline vs follow-up)		0.56 [0.22, 1.43]	0.38 [0.11, 1.32]
Ward			
A			1.00
B			0.60 [0.20, 1.83]
C			0.80 [0.21, 3.05]
D			0.75 [0.24, 2.35]
E			0.61 [0.19, 1.90]
F			0.23 [0.05, 1.02]
**Variance component estimates (95% CI)**			
Observation session level (n=120)	2.13 [1.25, 3.62]	2.09 [1.23, 3.55]	1.96 [1.14, 3.37]
Patient level (n=273)	0.51 [0.23, 1.13]	0.51 [0.23, 1.13]	0.51 0.23, 1.13]

Table 3 QuIS multilevel logistic regression results: odds ratios (OR) of a negative interaction

Table 4 shows the mean patient evaluations of emotional care (PEECH) values by experimental group. Higher scores indicate better patient-reported experiences. Connection subscale scores were consistently lower than on other subscales. Differences between groups at follow-up favor CLECC in total score and three of the four subscales, but these differences were not significant.

Levels of staff self-reported empathy using Jefferson Scale of Empathy varied across individual wards at baseline and at follow-up. There was no significant difference between groups (p=0.800).

At ward level, intracluster correlations (ICCs) for QuIS, PEECH and JSE were low (<0.027). The ICC for QuIS at ward level was higher, although still small (0.071), but high at observation session level (0.411).

For peer review only - http://bmjopen.bmj.com/site/about/guidelines.xhtml

PEECH<br><br>Mean (SD)	Baseline (n=168)		Follow up (n=186)		P value
	CLECC (n=105)	Control (n=63)	CLECC (n=123)	Control (n=63)	
Security (0 to 3)	2.48 (0.55)	2.36 (0.51)	2.48 (0.50)	2.46 (0.48)	0.653
Knowing (0 to 3)	2.18 (0.82)	2.30 (0.72)	2.19 (0.88)	2.26 (0.66)	0.800
Personal value (0 to 3)	2.34 (0.57)	2.35 (0.58)	2.43 (0.57)	2.31 (0.57)	0.071
Connection (0 to 3)	1.68 (0.74)	1.61 (0.84)	1.81 (0.82)	1.71 (0.63)	0.350
Total PEECH score (0 to 66)	49.2 (11.5)	48.4 (12)	50.6 (11.3)	48.5 (9.8)	0.116

Table 4 PEECH mean (SD) scores by experimental group (baseline and follow-up)

Discussion:

This study aimed to deliver a compassionate care intervention in acute care settings, pilot the use of experimental methodology and assess the performance of selected outcome measures. We aimed to provide an evidence base to guide future trial design and implementation, including acceptability of ward level randomisation, the feasibility of assessing outcome measures, and other measures of trial implementation such as recruitment and inclusivity, sample size calculation and clustering for future trial, blinding and contamination. The high recruitment rate of ward managers on behalf of their teams and subsequent lack of attrition of any of the ward teams recruited indicate that trial randomisation and the CLECC intervention are acceptable to medical and surgical nursing teams in acute care hospitals. Recruitment processes and methods appeared to be inclusive of all nursing staff levels and of older patients. Observations, in particular, were highly acceptable to patients with an overall recruitment rates of 93%. Questionnaire response rates varied, as discussed below. Our findings suggest that the CLECC intervention may have a favourable effect in reducing negative interactions between staff and patients, and in reducing patients' experiences of lack of emotional connection with staff. However as expected, because of the scale of this pilot, there is no certainty that any apparent positive effects are not produced by chance alone, rather than the impact of the CLECC intervention.

For peer review only - http://bmjopen.bmj.com/site/about/guidelines.xhtml

Hospitalised older patients with cognitive impairment are a traditionally hard-to-reach group and even though they appear more prone to negative experiences of hospital care,[28] they are often excluded from research.[5 29 30] It is estimated that up to 25% of beds in acute hospitals are occupied by people with dementia, with the figure likely to be higher on specialist older people's wards.[31 32] While cognitive deficits may limit some people's ability to share their experiences, our study has been successful in devising recruitment and data collection methods that maximise their inclusion. Overall 25% of patients observed in this study had evidence of cognitive impairment, suggesting a sample representative of the wider hospital population. Twelve percent of patient questionnaires returned were completed by patients with cognitive impairment, indicating the questionnaire method was less inclusive than observation methods. Participating in an observation does not require any particular state of health, abilities or performance form the patient in question, whereas participating in a questionnaire about one's care experiences requires a minimum orientation to place, language skills and attention.[30] In addition, using questionnaire methods may be psychologically threatening to patients still in receipt of care, regardless of cognitive status.[33]

The validity of observer ratings as accurate representation of patient experiences merits attention. Because main study observation and questionnaire data were gathered from different patient groups, it was not possible to test the validity of observer ratings against patient-reported experience. However, in earlier piloting work we found 79% agreement (weighted kappa 0.40: $P < 0.001$; indicating fair agreement) between patients' and observers' ratings of interaction quality.[22] Our earlier work did not include people with a cognitive impairment and validation of QuIS ratings with this patient group may be a necessary next step in the tool's development. In addition, if the proportion of negative interactions is the primary outcome measure in a future study, understanding which interactions are rated by observers (and, where possible, patients) as negative, and why, is an important next step, as is working with patient representatives to establish their views on the size of a meaningful reduction in negative interactions. Further study can also be used to develop more effective procedures to blind observers from experimental allocation in advance of an experimental study. In addition, the high intrucluster correlation we found at an observation session level merits the exploration of the cause of this variance and the

For peer review only - http://bmjopen.bmj.com/site/about/guidelines.xhtml

feasibility of different approaches to data collection that reduce its impact, for instance, shorter observation sessions. Our findings echo those of Goldberg et al.[29] that structured non-participant observation appears to be the most promising method to describe the experiences of older people with cognitive impairment in the general hospital setting, and so further evaluation and testing of QuIS across these parameters would be a valuable foundation to its further use as an outcome measure in acute settings.[29]

While the response rate to patient questionnaires was good (77%), of all the patient questionnaires returned, just 12% were completed by patients with cognitive impairment. While questionnaires provide an opportunity for patient to directly rate their care, less successful recruitment of a group known to be vulnerable to more negative experiences in hospital, means that any results may not be a valid representation of this group's experiences. The response rate to nursing questionnaires was low (36%), with some larger scale studies showing response rates of European nurses to be 62%, and US nurses to be around 39% .[34] Improving staff survey response rates through further feasibility work would improve confidence that conclusions in empathy levels across staff groups can be drawn with more confidence.

This study was piloted on a small number of wards in two hospitals so the findings are not generalisable. In addition, being observed could, in itself, change staff behaviours, and a common limitation of trials of this kind when it is not possible to conceal allocation from staff, is that bias may influence staff responses to observations and questionnaires. Additionally the finding of possible contamination between wards means that intervention and control conditions should not run in the same organisation over the same time period.

Findings from our wider study, reported elsewhere, that implementation of the CLECC intervention was uneven between wards, difficult to sustain and dependent on organisational support,[35] indicate that, while experimental research in this field is necessary, it will not provide sufficient explanation of results if conducted in isolation. However, the findings reported here represent valuable groundwork to the further development of sound experimental design in a field in which good design and implementation are very much needed.

For peer review only - http://bmjopen.bmj.com/site/about/guidelines.xhtml

Figure Legend: CONSORT flow diagram

For peer review only

For peer review only - http://bmjopen.bmj.com/site/about/guidelines.xhtml

Contributorship statement:

Lisa Gould: implementation, data collection, analysis, interpretation of results, write up of paper.
Jackie Bridges: design, implementation, data collection, analysis, interpretation of results, write up of paper.
Peter Griffiths: design, analysis, interpretation of results, and write up of paper.
Ruth Pickering: design, analysis, interpretation of results, and write up of paper.
Ines Mesa-Eguiagaray: analysis and interpretation of results, write up of paper.
Hannah Barker: implementation, data collection, analysis, interpretation of results, write up of paper.
Lisa Shipway: implementation, data collection, analysis, interpretation of results, write up of paper.
Paula Libberton: implementation, data collection, analysis, interpretation of results, write up of paper.

Acknowledgements

The authors thank all patients, research nurses and staff in the participating hospitals. Without their support, the study would not have been possible. With thanks to our fellow investigators not listed as authors:
Chable; Rosemary
Munro; Emma
Fuller; Alison
Raftery; James
Aihie Sayer; Avan
Westwood; Greta
Wigley; Wendy
Yao; Guiqing

Funding Acknowledgement:
This project was funded by the National Institute for Health Research [Health Services and Delivery Research programme] (project number 13/07/48)

Department of Health Disclaimer:
The views and opinions expressed therein are those of the authors and do not necessarily reflect those of the Health Services and Delivery Research programme, NIHR, NHS or the Department of Health.

For peer review only

References

1. Christensen K, Doblhammer G, Rau R, et al. Ageing populations: the challenges ahead. *Lancet* 2009;374(9696):1196-208. doi: 10.1016/S0140-6736(09)61460-4
2. Huang HT, Chang CM, Liu LF, et al. Trajectories and predictors of functional decline of hospitalised older patients. *J Clin Nurs* 2013;22(9-10):1322-31. doi: 10.1111/jocn.12055
3. Dent E, Chapman I, Howell S, et al. Frailty and functional decline indices predict poor outcomes in hospitalised older people. *Age Ageing* 2014;43(4):477-84. doi: 10.1093/ageing/aft181
4. Hickman LD, Phillips JL, Newton PJ, et al. Multidisciplinary team interventions to optimise health outcomes for older people in acute care settings: A systematic review. *Arch Gerontol Geriatr* 2015;61(3):322-9. doi: 10.1016/j.archger.2015.06.021
5. Bridges J, Flatley M, Meyer J. Older people's and relatives' experiences in acute care settings: systematic review and synthesis of qualitative studies. *International Journal of Nursing Studies* 2010;47(1):89-107.
6. Francis R. Independent Inquiry into care provided by Mid Staffordshire NHS Foundation Trust January 2005 – March 2009. https://www.gov.uk/government/uploads/system/uploads/attachment_data/file/279109/0375_i.pdf [Accessed 18.02.2017] London: The Stationary Office 2010.
7. Francis R. Report of the Mid Staffordshire NHS Foundation Trust public inquiry. http://webarchive.nationalarchives.gov.uk/20150407084003/http://www.midstaffspublicinquiry.com/report [Accessed 18.02.2017] London, 2013.
8. Care Quality Commission. Dignity and nutrition inspection programme: national overview. https://www.cqc.org.uk/sites/default/files/documents/20111007_dignity_and_nutrition_inspection_report_final_update.pdf [Accessed 18.02.2017] Newcastle upon Tyne: Care Quality Commission, 2011.
9. Chochinov H. Dignity and the essence of medicine: the A, B, C, and D of dignity conserving care. *British Medical Journal* 2007;335:184-87.
10. Hartrick G. Relational capacity: the foundation for interpersonal nursing practice. *Journal of Advanced Nursing* 1997;26(3):523-28.
11. Blomberg K, Griffiths P, Wengstrom Y, et al. Interventions for compassionate nursing care: a systematic review. *International Journal of Nursing Studies* 2016;62:137-55.
12. Barker H, Griffiths P, Gould L, et al. Quantity and quality of interaction between staff and older patients in UK hospital wards: a descriptive study. *International Journal of Nursing Studies* 2016;62:100-07.
13. StataCorp. Stata Statistical Software: Release 12. College Station, TX: StataCorp LP, 2011.
14. Fuller A, Unwin L. Expansive learning environments: integrating organizational and personal development. In: Rainbird H, Fuller A, Munro A, eds. Workplace learning in context. London: Routledge 2004:126-44.
15. Bridges J, Fuller A. Creating Learning Environments for Compassionate Care (CLECC): a programme to promote compassionate care by health and social care teams. *International Journal of Older People Nursing* 2015;10(1):48-58.
16. Fuller A. Theories of learning and communities of practice. In: Hughes J, Jewson N, Unwin L, eds. Learning in communities of practice. London: Routledge 2007.
17. Fuller A, Unwin L. Expansive learning environments: integrating organisational and personal development. In: Rainbird H, Fuller A, Munro A, eds. Workplace learning in context. London: Routledge 2004:126-44.
18. Wenger E. Communities of practice: learning, meaning and identity. Cambridge: Cambridge University Press 1998.
19. May C, Finch T, Mair F, et al. Understanding the implementation of complex interventions in health care: the normalization process model. *BMC Health Services Research* 2007;7(1):148.

For peer review only - http://bmjopen.bmj.com/site/about/guidelines.xhtml

20. Bridges J, Fuller A. Creating learning environments for compassionate care: a programme to promote compassionate care by health and social care teams. *Int J Older People Nurs* 2015;10(1):48-58. doi: 10.1111/opn.12055
21. Dean R, Proudfoot R, Lindesay J. The quality of interactions schedule(QUIS): development, reliability and use in the evaluation of two domus units. *International Journal of Geriatric Psychiatry* 1993;8:819-26.
22. McLean C, Griffiths P, Mesa-Eguiagaray I, et al. Reliability, feasibility, and validity of the quality of interactions schedule (QuIS) in acute hospital care: an observational study. *BMC Health Serv Res* 2017;17(1):380. doi: 10.1186/s12913-017-2312-2
23. Murrells T, Robert G, Adams M, et al. Measuring relational aspects of hospital care in England with the 'Patient Evaluation of Emotional Care during Hospitalisation'(PEECH) survey questionnaire. *BMJ Open* 2013;3(1)
24. Williams A, Dawson S, Kristjanson L. Translating theory into practice: using action research to introduce a coordinated approach to emotional care. *Patient Education and Counseling* 2008;73(1):82-90.
25. Hojat M, Mangione S, Nasca TJ, et al. The Jefferson Scale of Physician Empathy: development and preliminary psychometric data. *Educational and Psychological Measurement* 2001;61(2):349-65.
26. Hojat M, Vergare MJ, Maxwell K, et al. The devil is in the third year: a longitudinal study of erosion of empathy in medical school. *Academic Medicine* 2009;84(9):1182-91.
27. Hojat M, Mangione S, Nasca TJ, et al. An empirical study of decline in empathy in medical school. *Medical Education* 2004;38(9):934-41.
28. Hartley P, Gibbins N, Saunders A, et al. The association between cognitive impairment and functional outcome in hospitalised older patients: a systematic review and meta-analysis. *Age Ageing* 2017 doi: 10.1093/ageing/afx007
29. Goldberg SE, Harwood RH. Experience of general hospital care in older patients with cognitive impairment: are we measuring the most vulnerable patients' experience? *BMJ Quality & Safety* 2013;22(12):977-80.
30. van Baalen A, Vingerhoets AJJM, Sixma HJ, et al. How to evaluate quality of care from the perspective of people with dementia: an overview of the literature. *Dementia* 2010;10(1):112-37.
31. Alzheimers Society. Counting the cost: Caring for people with dementia on hospital wards. 2009
32. Mukadam N, Sampson EL. A systematic review of the prevalence, associations and outcomes of dementia in older general hospital inpatients. *International Psychogeriatrics* 2011;23(3):344-55.
33 La Monica EL, Wolf RM, Madea AR, et al. Empathy and nursing care outcomes. *Scholarly Inquiry for Nursing Practice* 1987;1(3):197-213.
34. Aiken LH, Sermeus W, Van den Heede K, et al. Patient safety, satisfaction, and quality of hospital care: cross sectional surveys of nurses and patients in 12 countries in Europe and the United States. *BMJ: British Medical Journal* 2012;344
35. Bridges J, May C, Fuller A, et al. Optimising impact and sustainability: a qualitative process evaluation of a complex intervention targeted at compassionate care. *BMJ Qual Saf* 2017 doi: 10.1136/bmjqs-2017-006702

For peer review only - http://bmjopen.bmj.com/site/about/guidelines.xhtml

CONSORT Participant Flow Diagram for Pilot Cluster RCT

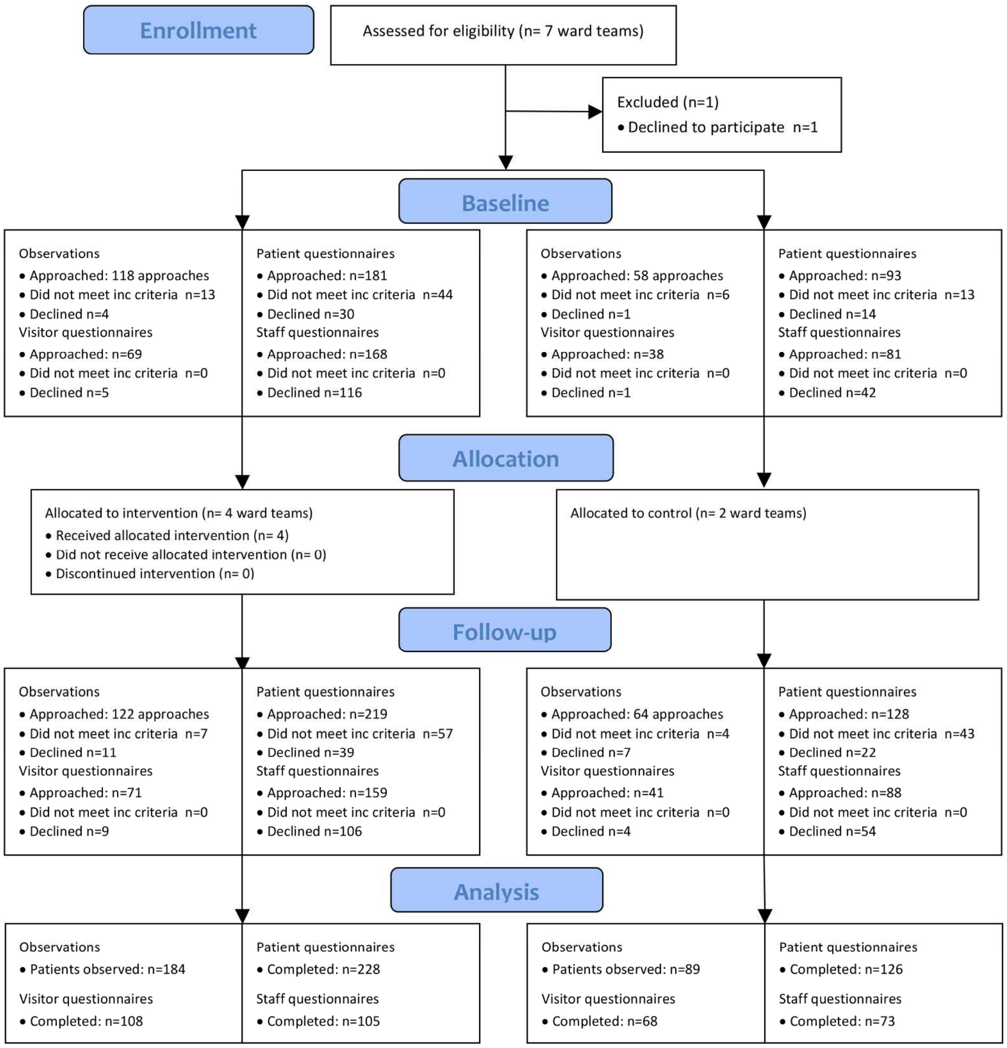

187x222mm (300 x 300 DPI)

Table 2
CONSORT checklist of information to include when reporting a pilot trial

Section/topic and item No	Standard checklist item	Extension for pilot trials	Page No where item is reported
Title and abstract			
1a	Identification as a randomised trial in the title	Identification as a pilot or feasibility randomised trial in the title	Title (not allocated page number yet)
1b	Structured summary of trial design, methods, results, and conclusions (for specific guidance see CONSORT for abstracts)	Structured summary of pilot trial design, methods, results, and conclusions (for specific guidance see CONSORT abstract extension for pilot trials)	Abstract (not allocated page number yet)
Introduction			
Background and objectives:			
2a	Scientific background and explanation of rationale	Scientific background and explanation of rationale for future definitive trial, and reasons for randomised pilot trial	Pg 1-2
2b	Specific objectives or hypotheses	Specific objectives or research questions for pilot trial	Pg 2
Methods			
Trial design:			
3a	Description of trial design (such as parallel, factorial) including allocation ratio	Description of pilot trial design (such as parallel, factorial) including allocation ratio	Pg 2 and 3
3b	Important changes to methods after trial commencement (such as eligibility criteria), with	Important changes to methods after pilot trial commencement (such as eligibility criteria), with reasons	N/A, no changes made

For peer review only

Section/topic and item No	Standard checklist item	Extension for pilot trials	Page No where item is reported
	reasons		
Participants:			
4a	Eligibility criteria for participants		Pg 4 and 5
4b	Settings and locations where the data were collected		Pg 2
4c		How participants were identified and consented	Pg 3, 4 and 5
Interventions:			
5	The interventions for each group with sufficient details to allow replication, including how and when they were actually administered		Pg 3
Outcomes:			
6a	Completely defined prespecified primary and secondary outcome measures, including how and when they were assessed	Completely defined prespecified assessments or measurements to address each pilot trial objective specified in 2b, including how and when they were assessed	Pg 3, 4 and 5
6b	Any changes to trial outcomes after the trial commenced, with reasons	Any changes to pilot trial assessments or measurements after the pilot trial commenced, with reasons	N/A no changes
6c		If applicable, prespecified criteria used to judge whether, or how, to proceed with future definitive trial	

For peer review only

Section/topic and item No	Standard checklist item	Extension for pilot trials	Page No where item is reported
Sample size:			
7a	How sample size was determined	Rationale for numbers in the pilot trial	Pg 4
7b	When applicable, explanation of any interim analyses and stopping guidelines		N/A
Randomisation:			
Sequence generation:			
8a	Method used to generate the random allocation sequence		Pg 2 and 3 (ward randomisation) and 4 (observation randomisation)
8b	Type of randomisation; details of any restriction (such as blocking and block size)	Type of randomisation(s); details of any restriction (such as blocking and block size)	Pg 2 and 3, and 4
Allocation concealment mechanism:			
9	Mechanism used to implement the random allocation sequence (such as sequentially numbered containers), describing any steps taken to conceal the sequence until interventions were assigned		Pg 2 and 3,4 and 5
Implementation:			
10	Who generated the random allocation sequence, enrolled participants, and assigned participants to interventions		Pg 2, 3, 4 and 5

For peer review only

Section/topic and item No	Standard checklist item	Extension for pilot trials	Page No where item is reported
Blinding:			
11a	If done, who was blinded after assignment to interventions (eg, participants, care providers, those assessing outcomes) and how		Pg 5
11b	If relevant, description of the similarity of interventions		N/A
Analytical methods:			
12a	Statistical methods used to compare groups for primary and secondary outcomes	Methods used to address each pilot trial objective whether qualitative or quantitative	6, 7, 8 and 9
12b	Methods for additional analyses, such as subgroup analyses and adjusted analyses	Not applicable	N/A
Results			
Participant flow (a diagram is strongly recommended):			
13a	For each group, the numbers of participants who were randomly assigned, received intended treatment, and were analysed for the primary outcome	For each group, the numbers of participants who were approached and/or assessed for eligibility, randomly assigned, received intended treatment, and were assessed for each objective	Figure 1, and pg 6, 7 and 8
13b	For each group, losses and exclusions after randomisation, together with reasons		Figure 1, and pg 6, 7 and 8

For peer review only

Section/topic and item No	Standard checklist item	Extension for pilot trials	Page No where item is reported
Recruitment:			
14a	Dates defining the periods of recruitment and follow-up		Pg 6
14b	Why the trial ended or was stopped	Why the pilot trial ended or was stopped	N/A
Baseline data:			
15	A table showing baseline demographic and clinical characteristics for each group		Table 1
Numbers analysed:			
16	For each group, number of participants (denominator) included in each analysis and whether the analysis was by original assigned groups	For each objective, number of participants (denominator) included in each analysis. If relevant, these numbers should be by randomised group	Figure 1, pg 6, 7 and 8
Outcomes and estimation:			
17a	For each primary and secondary outcome, results for each group, and the estimated effect size and its precision (such as 95% confidence interval)	For each objective, results including expressions of uncertainty (such as 95% confidence interval) for any estimates. If relevant, these results should be by randomised group	Pg 8 and 9. Tables 2, 3 and 4
17b	For binary outcomes, presentation of both absolute and relative effect sizes is recommended	Not applicable	N/A
Ancillary analyses:			

For peer review only

Section/topic and item No	Standard checklist item	Extension for pilot trials	Page No where item is reported
18	Results of any other analyses performed, including subgroup analyses and adjusted analyses, distinguishing prespecified from exploratory	Results of any other analyses performed that could be used to inform the future definitive trial	N/A
Harms:			
19	All important harms or unintended effects in each group (for specific guidance see CONSORT for harms)		No harms
19a		If relevant, other important unintended consequences	N/A
Discussion			
Limitations:			
20	Trial limitations, addressing sources of potential bias, imprecision, and, if relevant, multiplicity of analyses	Pilot trial limitations, addressing sources of potential bias and remaining uncertainty about feasibility	Pg 11
Generalisability:			
21	Generalisability (external validity, applicability) of the trial findings	Generalisability (applicability) of pilot trial methods and findings to future definitive trial and other studies	Pg 10 and 11
Interpretation:			
22	Interpretation consistent with results, balancing benefits and	Interpretation consistent with pilot trial objectives and findings,	Pg 9, 10 and 11

For peer review only

Section/topic and item No	Standard checklist item	Extension for pilot trials	Page No where item is reported
	harms, and considering other relevant evidence	balancing potential benefits and harms, and considering other relevant evidence	
22a		Implications for progression from pilot to future definitive trial, including any proposed amendments	Pg 10 and 11
Other information			
Registration:			
23	Registration number and name of trial registry	Registration number for pilot trial and name of trial registry	ISRCTN16789770
Protocol:			
24	Where the full trial protocol can be accessed, if available	Where the pilot trial protocol can be accessed, if available	Submitted as supplementary file at BMJ open
Funding:			
25	Sources of funding and other support (such as supply of drugs), role of funders		NIHR HS&DR Programme
26		Ethical approval for the study was granted by the national Social Care Research Ethics Committee 14/IEC08/1018	

For peer review only
